# Cross-oncopanel study reveals high sensitivity and accuracy with overall analytical performance depending on genomic regions

**DOI:** 10.1186/s13059-021-02315-0

**Published:** 2021-04-16

**Authors:** Binsheng Gong, Dan Li, Rebecca Kusko, Natalia Novoradovskaya, Yifan Zhang, Shangzi Wang, Carlos Pabón-Peña, Zhihong Zhang, Kevin Lai, Wanshi Cai, Jennifer S. LoCoco, Eric Lader, Todd A. Richmond, Vinay K. Mittal, Liang-Chun Liu, Donald J. Johann, James C. Willey, Pierre R. Bushel, Ying Yu, Chang Xu, Guangchun Chen, Daniel Burgess, Simon Cawley, Kristina Giorda, Nathan Haseley, Fujun Qiu, Katherine Wilkins, Hanane Arib, Claire Attwooll, Kevin Babson, Longlong Bao, Wenjun Bao, Anne Bergstrom Lucas, Hunter Best, Ambica Bhandari, Halil Bisgin, James Blackburn, Thomas M. Blomquist, Lisa Boardman, Blake Burgher, Daniel J. Butler, Chia-Jung Chang, Alka Chaubey, Tao Chen, Marco Chierici, Christopher R. Chin, Devin Close, Jeffrey Conroy, Jessica Cooley Coleman, Daniel J. Craig, Erin Crawford, Angela del Pozo, Ira W. Deveson, Daniel Duncan, Agda Karina Eterovic, Xiaohui Fan, Jonathan Foox, Cesare Furlanello, Abhisek Ghosal, Sean Glenn, Meijian Guan, Christine Haag, Xinyi Hang, Scott Happe, Brittany Hennigan, Jennifer Hipp, Huixiao Hong, Kyle Horvath, Jianhong Hu, Li-Yuan Hung, Mirna Jarosz, Jennifer Kerkhof, Benjamin Kipp, David Philip Kreil, Paweł Łabaj, Pablo Lapunzina, Peng Li, Quan-Zhen Li, Weihua Li, Zhiguang Li, Yu Liang, Shaoqing Liu, Zhichao Liu, Charles Ma, Narasimha Marella, Rubén Martín-Arenas, Dalila B. Megherbi, Qingchang Meng, Piotr A. Mieczkowski, Tom Morrison, Donna Muzny, Baitang Ning, Barbara L. Parsons, Cloud P. Paweletz, Mehdi Pirooznia, Wubin Qu, Amelia Raymond, Paul Rindler, Rebecca Ringler, Bekim Sadikovic, Andreas Scherer, Egbert Schulze, Robert Sebra, Rita Shaknovich, Qiang Shi, Tieliu Shi, Juan Carlos Silla-Castro, Melissa Smith, Mario Solís López, Ping Song, Daniel Stetson, Maya Strahl, Alan Stuart, Julianna Supplee, Philippe Szankasi, Haowen Tan, Lin-ya Tang, Yonghui Tao, Shraddha Thakkar, Danielle Thierry-Mieg, Jean Thierry-Mieg, Venkat J. Thodima, David Thomas, Boris Tichý, Nikola Tom, Elena Vallespin Garcia, Suman Verma, Kimbley Walker, Charles Wang, Junwen Wang, Yexun Wang, Zhining Wen, Valtteri Wirta, Leihong Wu, Chunlin Xiao, Wenzhong Xiao, Shibei Xu, Mary Yang, Jianming Ying, Shun H. Yip, Guangliang Zhang, Sa Zhang, Meiru Zhao, Yuanting Zheng, Xiaoyan Zhou, Christopher E. Mason, Timothy Mercer, Weida Tong, Leming Shi, Wendell Jones, Joshua Xu

**Affiliations:** 1 Division of Bioinformatics and Biostatistics, National Center for Toxicological Research, US Food and Drug Administration, Jefferson, AR 72079 USA; 2 Immuneering Corporation, One Broadway, 14th Floor, Cambridge, MA 02142 USA; 3 Agilent Technologies, 11011 N Torrey Pines Rd, La Jolla, CA 92037 USA; 4 Department of Information Science, University of Arkansas at Little Rock, 2801 S. Univ. Ave, Little Rock, AR 72204 USA; 5 State Key Laboratory of Genetic Engineering, School of Life Sciences and Shanghai Cancer Hospital/Cancer Institute, Fudan University, Shanghai, 200438 China; 6 Agilent Technologies, 5301 Stevens Creek Blvd, Santa Clara, CA 95051 USA; 7 Research and Development, Burning Rock Biotech, Shanghai, 201114 China; 8 Bioinformatics, Integrated DNA Technologies, Inc., 1710 Commercial Park, Coralville, IA 52241 USA; 9 iGeneTech, 8 Shengmingyuan Rd., Zhongguancun Life Science Park, Changping District, Beijing, 100080 China; 10 Illumina Inc., 5200 Illumina Way, San Diego, CA 92122 USA; 11 Research and Development, QIAGEN Sciences Inc., Frederick, MD 21703 USA; 12 Market & Application Development Bioinformatics, Roche Sequencing Solutions Inc., 4300 Hacienda Dr, Pleasanton, CA 94588 USA; 13 Thermo Fisher Scientific, 110 Miller Ave, Ann Arbor, MI 48104 USA; 14 Clinical Diagnostic Division, Thermo Fisher Scientific, 46500 Kato Rd, Fremont, CA 94538 USA; 15 Winthrop P Rockefeller Cancer Institute, University of Arkansas for Medical Sciences, 4301 W Markham St, Little Rock, AR 72205 USA; 16 Departments of Medicine, Pathology, and Cancer Biology, College of Medicine and Life Sciences, University of Toledo Health Sciences Campus, 3000 Arlington Ave, Toledo, OH 43614 USA; 17 National Institute of Environmental Health Sciences, Research Triangle Park, NC 27709 USA; 18 Department of Immunology, Genomics and Microarray Core Facility, University of Texas Southwestern Medical Center, 5323 Harry Hine Blvd, Dallas, TX 75390 USA; 19 Research and Development, Roche Sequencing Solutions Inc., 500 South Rosa Rd, Madison, WI 53719 USA; 20 Clinical Sequencing Division, Thermo Fisher Scientific, 180 Oyster Point Blvd, South San Francisco, CA 94080 USA; 21 Marketing, Integrated DNA Technologies, Inc., 1710 Commercial Park, Coralville, IA 52241 USA; 22 Icahn Institute and Dept. of Genetics and Genomic Sciences Icahn School of Medicine at Mount Sinai, 1425 Madison Ave, New York, NY 10029 USA; 23 Greenwood Genetic Center, 106 Gregor Mendel Circle, Greenwood, SC 29646 USA; 24 Department of Pathology, Fudan University Shanghai Cancer Center, Fudan University, Shanghai, 200032 China; 25 Department of Oncology, Shanghai Medical College, Fudan University, Shanghai, 200032 China; 26 Institute of Pathology, Fudan University, Shanghai, 200032 China; 27 JMP Life Sciences, SAS Institute Inc., Cary, NC 27519 USA; 28 Departments of Pathology and Pediatrics, University of Utah School of Medicine, Salt Lake City, UT 84108 USA; 29 R&D Genomics MPS, Institute for Clinical and Experimental Pathology ARUP Laboratories, 500 Chipeta Way, Salt Lake City, UT 84108 USA; 30 ResearchDx, Inc., 5 Mason, Irvine, CA 92618 USA; 31 Department of Computer Science, Engineering and Physics, University of Michigan-Flint, Flint, MI 48502 USA; 32 Garvan Institute of Medical Research, Sydney, NSW Australia; 33 St Vincent’s Clinical School, University of New South Wales, Sydney, NSW 2010 Australia; 34 Department of Pathology, College of Medicine and Life Sciences, The University of Toledo, Toledo, OH 43614 USA; 35 Lucas County Coroner’s Office, 2595 Arlington Ave., Toledo, OH 43614 USA; 36 Division of Gastroenterology and Hepatology, Mayo Clinic, Rochester, MN 55905 USA; 37 OmniSeq, Inc. 700 Ellicott St, Buffalo, NY 14203 USA; 38 Department of Physiology and Biophysics, Weill Cornell Medicine, Cornell University, New York, NY 10065 USA; 39 Stanford Genome Technology Center, Stanford University, Palo Alto, CA 94304 USA; 40 Division of Genetic and Molecular Toxicology, National Center for Toxicological Research, US Food and Drug Administration, Jefferson, AR 72079 USA; 41 Fondazione Bruno Kessler, 38123 Trento, Italy; 42 Department of Medicine, College of Medicine and Life Sciences, The University of Toledo, Toledo, OH 43614 USA; 43 Institute of Medical and Molecular Genetics (INGEMM), Hospital Universitario La Paz, CIBERER Instituto de Salud Carlos III, 28046 Madrid, Spain; 44 EATRIS ERIC- European Infrastructure for Translational Medicine, De Boelelaan 1118, 1081 HZ Amsterdam, The Netherlands; 45 Kinghorn Centre for Clinical Genomics, Garvan Institute of Medical Research, Sydney, NSW Australia; 46 St Vincent’s Clinical School, Faculty of Medicine, University of New South Wales, Sydney, NSW Australia; 47 Cancer Genetics Inc, 201 Route 17 N, Meadows Office Building, Rutherford, NJ 07070 USA; 48 Institute for Personalized Cancer Therapy, MD Anderson Cancer Center, 6565 MD Anderson Blvd, Houston, TX 77030 USA; 49 Pharmaceutical Informatics Institute, College of Pharmaceutical Sciences, Zhejiang University, Hangzhou, 310058 Zhejiang China; 50 HK3 Lab, Milan, Italy; 51 Molecular Laboratory, Prof. F. Raue, Im Weiher 12, Heidelberg, Germany; 52 Agilent Technologies, 1834 State Hwy 71 West, Cedar Creek, TX 78612 USA; 53 Department of Pathology, Strata Oncology, Inc., Ann Arbor, MI 48103 USA; 54 Human Genome Sequencing Center, Baylor College of Medicine, 1 Baylor Plaza, Houston, TX 77030 USA; 55 Massachusetts General Hospital, Harvard Medical School, Boston, MA 02114 USA; 56 NGS Products and Services, Integrated DNA Technologies, Inc., 1710 Commercial Park, Coralville, IA 52241 USA; 57 Molecular Genetics Laboratory, Molecular Diagnostics Division, London Health Sciences Centre, 800 Commissioners Rd E, London, Ontario N6A5W9 Canada; 58 Division of Anatomic Pathology, Mayo Clinic, 200 First Street SW, Rochester, MN 55905 USA; 59 Bioinformatics Research, Institute of Molecular Biotechnology, Boku University Vienna, Vienna, Austria; 60 Małopolska Centre of Biotechnology, Jagiellonian University, Krakow, Poland; 61 Department of Biotechnology, Boku University, Vienna, Austria; 62 Institute of Medical and Molecular Genetics (INGEMM), Hospital Universitario La Paz, IdiPaz, CIBERER Instituto de Salud Carlos III, 28046 Madrid, Spain; 63 ITHACA, European Reference Network on Rare Congenital Malformations and Rare Intellectual Disability, European Commission, Lille, France; 64 Department of Pathology, National Cancer Center/National Clinical Research Center for Cancer/Cancer Hospital, Chinese Academy of Medical Sciences, No.17, Panjiayuan Nanli, Chaoyang District, Beijing, 100021 China; 65 Center of Genome and Personalized Medicine, Institute of Cancer Stem Cell, Dalian Medical University, Dalian, 116044 Liaoning China; 66 Geneis, 5 Guangshun North St., Chaoyang District, Beijing, 100102 China; 67 GeneSmile Ltd Co., Jiangsu Cancer Hospital, 42 Baiziting St., Xuanwu District, Nanjing, 210009 Jiangsu China; 68 Genycell Biotech España, Calle Garrido Atienza, 18320 Santa Fe, Granada, Spain; 69 CMINDS Research Center, Department of Electrical and Computer Engineering, College of Engineering, University of Massachusetts Lowell, Lowell, MA 01854 USA; 70 Department of Genetics, University of North Carolina, 250 Bell Tower Drive, Chapel Hill, NC 27599 USA; 71 Accugenomics, Inc., 1410 Commonwealth Drive, Suite 105, Wilmington, NC 20403 USA; 72 Translational Research Laboratory, Belfer Center for Applied Cancer Science, Dana-Farber Cancer Institute, 360 Longwood Ave, Boston, MA 02215 USA; 73 Bioinformatics and Computational Biology Laboratory, National Heart Lung and Blood Institute, National Institutes of Health, Bethesda, MD 20892 USA; 74 Astrazeneca Pharmaceuticals, 35 Gatehouse Dr, Waltham, MA 02451 USA; 75 Department of Pathology and Laboratory Medicine, Western University, London, Ontario N6A3K7 Canada; 76 Institute for Molecular Medicine Finland (FIMM), Nordic EMBL Partnership for Molecular Medicine, HiLIFE Unit, Biomedicum Helsinki 2U (D302b), P.O. Box 20, (Tukholmankatu 8), FI-00014 University of Helsinki, Helsinki, Finland; 77 Laboratory for Molecular Genetics, Endocrine Practice, Im Weiher 12, 69121 Heidelberg, Germany; 78 Division of Systems Biology, National Center for Toxicological Research, US Food and Drug Administration, Jefferson, AR 72079 USA; 79 Center for Bioinformatics and Computational Biology, and the Institute of Biomedical Sciences, School of Life Sciences, East China Normal University, 500 Dongchuan Rd, Shanghai, 200241 China; 80 National Centre for Cardiovascular Research (CNIC), Madrid, Spain; 81 Primbio Genes Biotechnology, Building C6-501, Biolake, No.666 Gaoxin Ave., East Lake High-tech Development Zone, Wuhan, 430074 Hubei China; 82 National Center for Biotechnology Information, National Library of Medicine, National Institutes of Health, 8600 Rockville Pike, Bethesda, MD 20894 USA; 83 Center of Molecular Medicine, Central European Institute of Technology, Masaryk University, Kamenice 5, 625 00 Brno, Czech Republic; 84 Center for Genomics, School of Medicine, Loma Linda University, Loma Linda, CA 92350 USA; 85 Division of Microbiology & Molecular Genetics, Department of Basic Sciences, School of Medicine, Loma Linda University, Loma Linda, CA 92350 USA; 86 Center for Individualized Medicine, Mayo Clinic, Scottsdale, AZ 85259 USA; 87 Department of Health Sciences, Mayo Clinic, Scottsdale, AZ 85259 USA; 88 Department of Molecular Pharmacology and Experimental Therapeutics, Mayo Clinic, Scottsdale, AZ 85259 USA; 89 College of Chemistry, Sichuan University, Chengdu, 610064 Sichuan China; 90 Science for Life Laboratory, Karolinska Institutet, Tomtebodavägen 23B, 171 65 Solna, Sweden; 91 National Center for Biotechnology Information, National Library of Medicine, National Institutes of Health, 45 Center Drive, Bethesda, MD 20894 USA; 92 Department of Biostatistics, Columbia Mailman School of Public Health, 722 West 168th St., New York, NY 10032 USA; 93 Center for Genomic Sciences, LKS Faculty of Medicine, The University of Hong Kong, Hong Kong, SAR China; 94 Clinical Laboratory, Burning Rock Biotech, Guangzhou, 510300 Guangdong China; 95 Geneplus, PKUCare Industrial Park, Changping District, Beijing, 102206 China; 96 Australian Institute of Bioengineering and Nanotechnology, University of Queensland, Brisbane, QLD Australia; 97 Genomics and Epigenetics Theme, Garvan Institute of Medical Research, Sydney, NSW Australia; 98 Human Phenome Institute, Fudan University, Shanghai, 201203 China; 99 Fudan-Gospel Joint Research Center for Precision Medicine, Fudan University, Shanghai, 200438 China; 100 Q2 Solutions - EA Genomics, 5927 S Miami Blvd, Morrisville, NC 27560 USA

**Keywords:** Oncopanel sequencing, Target enrichment, Molecular diagnostics, Reproducibility, Analytical performance, Precision medicine

## Abstract

**Background:**

Targeted sequencing using oncopanels requires comprehensive assessments of accuracy and detection sensitivity to ensure analytical validity. By employing reference materials characterized by the U.S. Food and Drug Administration-led SEquence Quality Control project phase2 (SEQC2) effort, we perform a cross-platform multi-lab evaluation of eight Pan-Cancer panels to assess best practices for oncopanel sequencing.

**Results:**

All panels demonstrate high sensitivity across targeted high-confidence coding regions and variant types for the variants previously verified to have variant allele frequency (VAF) in the 5–20% range. Sensitivity is reduced by utilizing VAF thresholds due to inherent variability in VAF measurements. Enforcing a VAF threshold for reporting has a positive impact on reducing false positive calls. Importantly, the false positive rate is found to be significantly higher outside the high-confidence coding regions, resulting in lower reproducibility. Thus, region restriction and VAF thresholds lead to low relative technical variability in estimating promising biomarkers and tumor mutational burden.

**Conclusion:**

This comprehensive study provides actionable guidelines for oncopanel sequencing and clear evidence that supports a simplified approach to assess the analytical performance of oncopanels. It will facilitate the rapid implementation, validation, and quality control of oncopanels in clinical use.

**Supplementary Information:**

The online version contains supplementary material available at 10.1186/s13059-021-02315-0.

## Background

Despite ongoing advances in sequencing technologies to date, the promise of their use for precision medicine has not yet borne out entirely for the majority of cancer patients. Although cancer is a genomic disease, most malignant tumors are currently classified and often treated based on the tissue of origin and the TNM (tumor, node, and metastases) staging system for solid tumors rather than presence or absence of relevant mutations in key oncogenic pathways. Presently, only a small number of specific mutations in genes such as *EGFR*, *ALK* [1], and *BRAF* [2] are routinely tested to inform clinical decisions. To move precision medicine from theoretical discussions to the bedside of individual patients, it requires accurate and reproducible testing of a broad spectrum of clinically relevant mutations combined with appropriate therapies. Hence, improved mutation testing is greatly needed to confidently classify individual tumors, reliably provide personalized therapeutic choices, and inform patient prognosis.

Next-generation sequencing (NGS) of targeted genomic regions has become increasingly common because targeted panels focus sequencing coverage on genomic regions more likely to harbor important genomic aberrations and thereby increase diagnostic sensitivity [3]. Oncopanels have several potential clinical applications, including identification of driver and potential driver mutations. Variants can be prioritized for their biological significance and cross-referenced with potentially useful targeted therapies. Some oncopanels are also used to assess micro-satellite instability [4], homologous recombination defect status [5], copy number alterations [6], and gene rearrangements [7]. There is increasing evidence that a significant proportion of patients with low-frequency alleles of these clinically actionable mutations show clinical response to targeted therapies [8]. Because key therapeutic decisions are based on these results, including choice of molecular-targeted therapy and prediction of outcomes, it is critical that mutation detection displays high sensitivity and low false positive (FP) rate and that reports to physicians are highly accurate [9–11]. Importantly, both oncopanels and downstream bioinformatic methods must be validated to be accurate, informative, and reliable.

Certain tumors that are genetically unstable may result in a large number of cellular proteins having somatic mutations (also known as neoantigens). More exposure of neoantigens increases the likelihood of non-self recognition by the adaptive immune system. If such a recognition event has occurred, but the tumor still progresses, a possible reason is the autoregulatory process of immune checkpoints which ordinarily prevent autoimmunity by deactivating active T cells in the tumor microenvironment. Therefore, if oncopanels are large enough in size, they can be used to estimate tumor mutational burden (TMB), an emerging indicator for immune checkpoint inhibitors in certain tumor types [12, 13].

In current practice, there are multiple Clinical Laboratory Improvement Amendments (CLIA)-certified labs that have implemented oncopanels for tumor profiling. At present, four pan-cancer panels have been approved or cleared by U.S. Food and Drug Administration (FDA): FoundationOne CDx [14], MSK- IMPACT [10], NantHealth Omics Core [15], and PGDx elio tissue complete [16]. In 2017, the FDA approved Merck’s Keytruda (pembrolizumab) for any unresectable or metastatic solid tumor with evidence of mismatch repair deficiency. Thus, this was the first approval that was biomarker-based and tissue agnostic [17]. The FDA accelerated the approval of Bayer’s Vitrakvi in 2018, and Roche’s Rozlytrek (entrectinib) in 2019, both of which are biomarker-based cancer treatments [18]. However, only a minority of cancer patients undergo high-throughput mutational profiling [19] and many physicians remain skeptical of the rigorousness of genomic testing [20], hesitant to deplete precious tumor material, and/or apprehensive about performing an additional tumor biopsy [21]. For oncopanel sequencing to become a useful and widely adopted clinical test, a thorough understanding of its sensitivity, reproducibility, and accuracy is greatly needed. To date, no study has assessed how distinct oncopanel methodologies perform with respect to variant allele frequency (VAF) and variant location, impacting sensitivity, FP rate, reproducibility, and potential biomarkers such as TMB for oncopanels. Previous studies comparing oncopanels have not used reference materials as test samples with (1) enough VAF range and sufficient diversity of mutations to thoroughly interrogate a sufficient number of clinically relevant mutations [22] and (2) sufficient material readily available for the scientific community [23–25]. In contrast, this study used recently characterized reference samples that contain an extensive collection of known positive and negative variants [26] to provide a much more definitive and comprehensive understanding of key variables that impact performance of oncopanels.

The FDA-led SEquencing and Quality Control project phase 2 (SEQC2) continues earlier consortium efforts [27–30] to advance best practices and address the technical gaps in genomics technologies. Its Somatic Mutation Working Group has extensively identified variant content of an individual pair of reference samples (breast cancer cell line HCC1395 and its normal counterpart) and established best practices for genome-wide somatic mutation calling [31, 32]. In parallel, the Oncopanel Sequencing Working Group’s efforts, which are detailed herein, utilize a recently identified novel set of comprehensive variants and negatives [26] from additional reference samples to compare variant calling across eight distinct oncopanels. We assessed intra-lab and cross-lab reproducibility, sensitivity for known positives, and accuracy (i.e., FP rate) through known negatives across a range of VAFs and in selected important genomic regions. The relationships between analytical variables and TMB estimation were also established to inform the selection of oncopanel and harmonization of TMB measurements across test platforms, which are important needs [33] identified recently to advance the successful utilization of TMB in clinical practice. This study provided a comprehensive evaluation of eight oncopanels to inform best practice guidelines for oncopanel sequencing (Table 1).

**Table 1:** Summary of recommendations

Issue	Recommendation
**Reference material selection**	A reference material (or a set of reference materials) with a high density of known variants spanning a range of low allele frequencies (e.g., from above 1 to 20%) is needed to assess the analytical performance of oncopanels (Fig. 1a). Ideally, the reference material will encompass a diversity of simple and complex variants in different cancer-associated genes.
**Sensitivity**	Sensitivity was found to be high (> 96.5%) for variants previously verified to have variant allele frequency (VAF) greater than 5% (Fig. 2a). We recommend that a less stringent VAF threshold is used to achieve high sensitivity for variants of VAF below 5%.
**Spike-in**	Utilizing a sample spiked-in at a specified amount (e.g., 5%) can provide additional variants at known allele frequencies for analytical validation of oncopanels.
**Variant type**	The sensitivity for detecting insertion and deletion variants (indels) is typically more variable and poorer than single nucleotide variants (SNVs), and this difference becomes more pronounced at low VAFs (Fig. 3). We recommend that analytical validation of indels is performed independently to SNVs.
**Determining VAF threshold**	Reference materials can be used to establish an optimal VAF threshold that reduces false positives (FPs) and retains sensitivity. Indels and SNVs may require different VAF thresholds to optimize performance (Fig. 3b).
**Controlling the FP rate**	In applications where a minimal FP rate is required, raising the allele frequency threshold was effective at reducing FPs. The additional restriction of analysis to the consensus targeted regions (CTR) can further reduce FP rate.
**Genomic location**	Genomic location can impact the rate of FPs detected, and we recommend that analytical validation of panels is independently performed inside and outside of the CTR (Fig. 4a).
**Cross-lab reproducibility**	Measuring the analytical performance of a panel in multiple labs is critical to establish reproducibility (Fig. 4c).
**Estimating tumor mutational burden (TMB)**	TMB estimation should be confined to the CTR of each panel. Applying a minimal VAF threshold was helpful to reduce FPs and improve TMB evaluation (Fig. 5d).

## Results

### Overview of study design

**Fig. 1:**
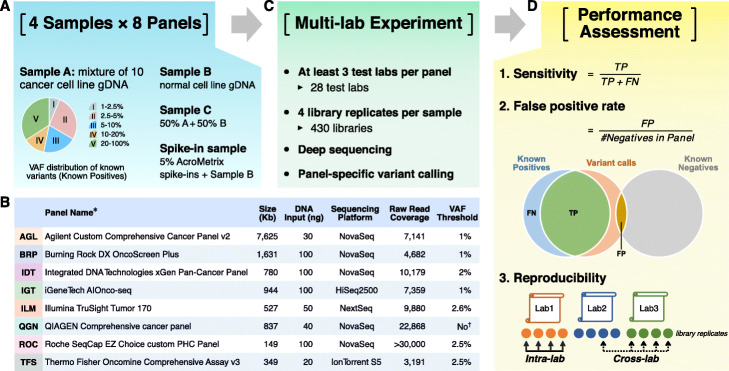
Comprehensive study design for assessing analytical performance of multiple pan-cancer targeted sequencing technologies. **a** Four samples were tested on 8 pan-cancer panels with at least 3 different test laboratories for each panel. **b** Basic information of 8 pan-cancer panels is listed in the embedded table (see Additional file 1: Table S1 for detailed information). *All participating panels are for research use only. ^†^QGN’s UMI-aware variant caller is able to call variants with VAF as low as 0.5%. **c** Each sample had 4 library replicates at each test laboratory. After sequencing, panel-specific variant calling was performed by each panel vendor. **d** Variant calling results were submitted for performance analysis including sensitivity, false positive call rate, and reproducibility

We employed four reference samples (Fig. 1a)—sample A (an equal mass pooled genomic DNA (gDNA) sample of ten cancer cell lines from the Agilent Universal Human Reference RNA (UHRR) material) [34], sample B (derived from a non-cancer male cell line), sample C (equal dilution of sample A and sample B), and a spike-in sample (sample B with 5% AcroMetrix hotspot synthetic controls [35]). The majority (about two thirds) of more than 42,000 known small variants (including SNVs, small indels, and multi-nucleotide variants (MNVs)) in the 22-Mb exon coding regions of sample A have a VAF below 20%, making sample A uniquely suitable for assessing the analytical performance of oncopanels. Sample C was included to increase the number of known variants with VAF between 1 and 2.5%. More details about the four reference samples are included in “Methods.” A total of eight oncopanels from eight distinct well-established panel providers were tested herein on these four samples, including Agilent Custom Comprehensive Cancer Panel v2 (AGL), Burning Rock DX OncoScreen Plus (BRP), Integrated DNA Technologies xGen Pan-Cancer Panel (IDT), iGeneTech AIOnco-seq (IGT), Illumina TruSight Tumor 170 (ILM), QIAGEN Comprehensive cancer panel (QGN), Roche SeqCap EZ Choice custom PHC Panel (ROC), and Thermo Fisher Oncomine Comprehensive Assay v3 (TFS). Vendors shipped the same reagent kits to all sites. Information about the oncopanels is included in Fig. 1b and Additional file 1: Table S1.

Each panel was tested in at least three independent laboratories with quadruplicates for each sample. In total, this unprecedented large-scale study encompassed 28 laboratories and 430 libraries (Fig. 1c). Each panel provider recruited the test laboratories and harmonized the experimental protocols to minimize cross-lab variations in the study. Libraries were sequenced on the same sequencing platform for each panel and the panel provider then performed panel-specific variant calling. For more information about sequencing and downstream bioinformatic pipelines, please see Additional file 2: Supplementary Methods. Variant calling results were submitted in variant call format (VCF) files, along with their targeted regions and whitelist/blacklist, to an independent group with sole knowledge of the known content of samples A, B, C, and the spike-in for performance evaluation (Fig. 1d). Intra-lab and cross-lab reproducibility were computed between library replicates as a measure of variant calling consistency. Sensitivity and FP rate were then estimated by utilizing the large sets of known positive (KP) and known negative (KN) loci from our companion work [26]. More than 42,000 KPs and 10 million KNs in sample A were characterized in the 22-Mb high-confidence exon coding regions through extensive sequencing of sample A and the individual cell line DNA samples that made up sample A. KN loci had the mismatch error rate below 0.25%. The distribution of KPs by VAF is shown in Fig. 1a.

### Sensitive detection of SNVs in the focused regions by all panels

**Fig. 2:**
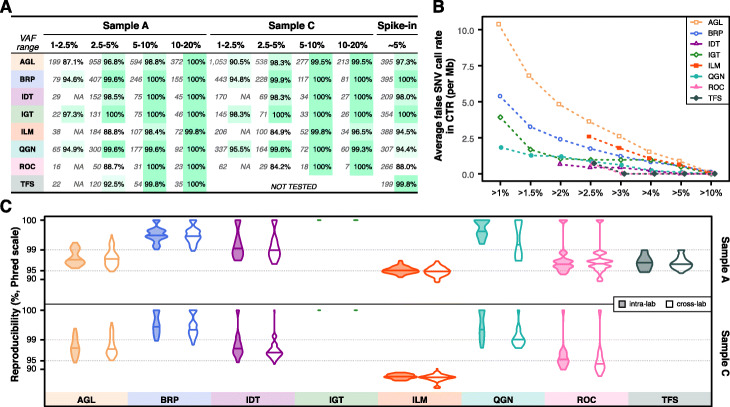
Reproducibility and sensitivity across VAF ranges for SNVs in the consensus targeted regions. **a** Table listed the number of known variants in each VAF range (left number), sensitivity (right number) for all 8 panels across all samples tested. For the panels with a built-in VAF threshold, “N/A” is listed if the VAF low bound is much lower than the panel provider’s chosen VAF threshold. The VAF threshold is 2.6% for ILM, 2.0% for IDT, 2.5% for ROC, and 2.5% for TFS, respectively. **b** Average false positive SNV calls per million across various VAF cutoffs. Jittering was applied to avoid overlapping. **c** Cross-lab and intra-lab reproducibility (in Phred scale) for variant calls with VAF between 2.5 and 20%

We evaluated the performance of detecting SNVs in the consensus targeted regions (CTR, see “Methods” for details) [26]. Given there is a general consensus that higher VAF variants are “easier” to detect, we focused our analysis on metrics that characterized performance relative to VAF, especially in the lower [1–10%] VAF range (Fig. 2). The number of KPs and observed sensitivity are listed side by side for each VAF range across three reference samples (A, C, and spike-in) and all panels (Fig. 2a). Sample C was not tested by TFS. Overall, sensitivity across all VAF ranges in all panels was high with narrow confidence intervals (Additional file 1: Table S2). It ranged from 87.1 to 98.3% for the lowest VAF range (1–2.5%) tested by AGL, BRP, IGT, and QGN (Fig. 2a). Sensitivity was greater than 96.8% for the 2.5–5% range and greater than 98.4% for the 5–20% range. The unusual sensitivity for ILM in sample C for the 10–20% range was due to a single false negative in all library replicates. Other exceptions were ILM, ROC, and TFS for the 2.5–5% range, as their variant calling pipelines had a VAF reporting threshold very close to 2.5%. So their sensitivity was reduced due to a boundary VAF threshold effect (see below for the effect of VAF cutoff on sensitivity). Accordingly, these threshold-dependent panels and IDT were not assessed for the lowest VAF range (since IDT’s pipeline utilizes a VAF reporting threshold of 2.0%). All false negatives in sample A or C were manually checked and can be explained by a combination of low VAF and the panel’s VAF threshold except the above mentioned single case for ILM in sample C. We initially anticipated similar sensitivity for the spike-in sample in the VAF range of 2.5–10%, as the variants were designed to be 5% VAF. However, this was not observed, likely due to interference of the nearby spike-in variants with amplicon primers, DNA capture, or variant calling. To derive sensitivity estimates, custom filtering of the spike-in’s variants was carried out by the panel providers to exclude variants under primer interference for QGN and TFS or variants in dense clusters for IDT (see Additional file 2: Supplementary Methods for details). Likewise, several dense variant clusters led to unexpectedly low reported sensitivity for ROC (88.0%).

All panels had FP rates lower than 10.5 per Mb in the CTR with each panel’s default VAF threshold, and the FP rates dropped as the VAF cutoff increased (Fig. 2b). The FP rate was noticeably different between panels for a VAF cutoff at 1%, but became increasingly similar as the VAF cutoff became more stringent. As a result, all panels had similar FP rates of approximately 1 FP per Mb or less for VAF greater than 5%. And all panels reported no FP calls for VAF greater than 10%. We also assessed both intra- and cross-lab reproducibility for variant calls in the CTR with VAF between 2.5 and 20% and observed that they were similar for each panel sample combination (Fig. 2c, Additional file 3: Fig. S1A), with cross-lab reproducibility being slightly worse than intra-lab reproducibility in a few instances. Results for sample A and sample C were similar for VAF above 5% but showed some noticeable differences in the VAF range 2.5–5%. The differences may be driven by VAF reporting boundary effects, as sample C has more variants in the lower VAF ranges (thus, near the VAF reporting thresholds for several panels) (Additional file 3: Fig. S1B).

### Impact of variant type, VAF cutoff, and genomic region on sensitivity

**Fig. 3:**
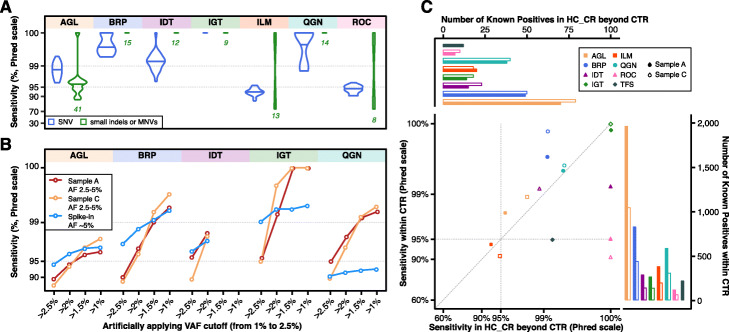
Impact of VAF cutoff, variant type, and genomic region on sensitivity (in Phred scale). **a** Violin distribution plots of estimated sensitivity for each panel in all sample A and C libraries for known SNVs (in blue on the left side) and other variants (small indels or MNVs, in green on the adjacent right side) with VAF between 2.5 and 20%. Total numbers of small indels and MNVs are listed under the corresponding violin plot. **b** Artificial VAF filters reduce sensitivity for known positives with VAF between 2.5 and 5% due to the variable VAF measurements. **c** High concordance of sensitivity in and outside of CTR (more specifically, in HC_CR beyond CTR) for known positives with VAF between 2.5 and 20%. Jittering was applied to one dot at the top right corner to avoid overlapping

We explored the impact of variant type on sensitivity for each panel by aggregating all sample A and C libraries (Fig. 3a). Non-SNV variant calling (small indels and multi-nucleotide variants (MNVs)) had more variable sensitivity than SNV variant calling for AGL, ILM, and ROC panels, as the number of known non-SNV small variants per panel in samples A and C is typically small by absolute numbers (green numbers in Fig. 3a, Additional file 3: Fig. S2). Thus the confidence interval was usually wider for non-SNV small variants (Additional file 1: Table S3). We characterized the impact of VAF on sensitivity comparing SNVs against non-SNV variants (Additional file 3: Fig. S2). For sample A, the difference between SNVs and non-SNV variants was most pronounced in the lowest VAF range. For both SNVs and non-SNV variants, sensitivity was generally observed to improve with known variants of higher expected VAFs.

We also applied a range of artificial VAF cutoffs in addition to each panel’s normal reporting thresholds from stringent (2.5%) to less stringent (1.5%) to better understand the impact of a VAF reporting threshold on sensitivity in detecting known positives in the VAF range 2.5–5% (Fig. 3b, see Additional file 1: Table S4 for confidence intervals). As the cutoff filter was more relaxed, and the boundary effects decreased, sensitivity rose expectedly across most panels and all reference samples. This increased sensitivity was more prominent in samples A and C, which may be explained by the fixed and higher VAF (5%) for variants in the spike-in sample. The boundary effects became negligible for the higher VAF ranges, i.e., 5–10% and 10–20%, in samples A and C (Additional file 1: Table S5).

Sensitivity within the CTR and outside of the CTR, but inside the High Confidence Coding Region (HC_CR, see “Methods” for details), was compared (Fig. 3c). For samples A and C, most panels performed consistently within and outside of the CTR (but within HC_CR). TFS, ILM (for sample C), and ROC had higher observed sensitivity outside of the CTR, but also had relatively few KPs in this region (Fig. 3c top bar graph). Thus narrower confidence intervals for sensitivity were achieved within the CTR (Additional file 1: Table S6).

### False positive rates were significantly higher beyond the focused regions

We employed three different methods to estimate the FP rate. Method 1 relies on the KNs (“via_KN”) in samples A and C (Additional file 3: Fig. S3A). Our list of KNs is extensive (> 10 M) but only available within the CTR (which is the majority of the coding region) and comprises about 30% of all coding regions. Using Method 1, a FP is a variant call on a KN position. A stringent consensus across multiple sequencing experiments was employed to determine the partial list of negatives within the CTR [26]. Method 1 may tend to underestimate the overall FP rate across all positions of the CTR. Method 2 marked any call in the normal sample B with VAF between 1 and 10% as a false positive (“B_low,” Additional file 3: Fig. S3B) due to strong evidence of this sample having normal diploid cell characteristics [26]. Method 3 leveraged sample mixture to differentiate FPs. Any variant call in sample C was deemed an FP if it was not called in any of four sample B libraries with VAF above 10% or any of four sample A libraries at the same test laboratory (“C_only,” Additional file 3: Fig. S3C). Method 2 might overestimate the FP rate by failing to exclude rare mosaic variants caused by cell line drift. Method 3 could underestimate the FP rate by failing to recognize those FP calls due to systematic errors as they would be called in all three samples. These three methods were compared and found to produce largely similar estimates within the CTR (Additional file 3: Fig. S3D), with the “B_low” method in one specific VAF range of IGT and ILM being the only exception. As expected, almost all FP calls within the CTR were irreproducible across library replicates for any panel, sample, and FP method (Additional file 3: Fig. S4). Those few exceptions were then confirmed through manual curation as systematic errors (see the legend for Additional file 3: Fig. S4).

**Fig. 4:**
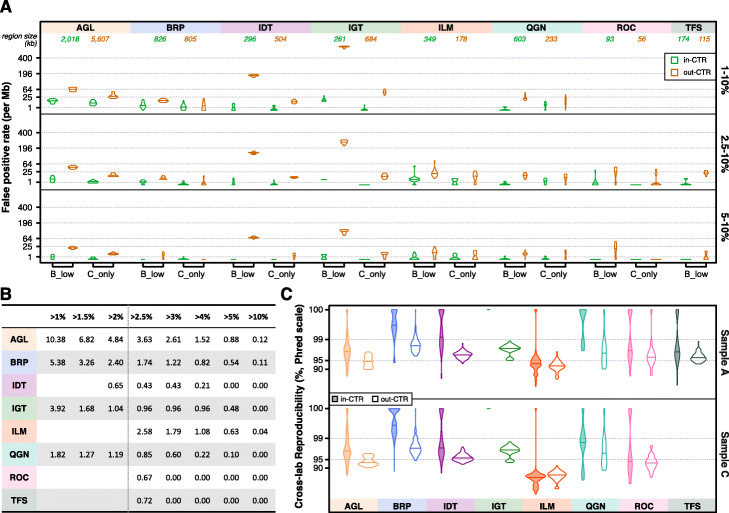
Impact of CTR region on FP rate and reproducibility. **a** FP rate in and outside of the CTR using two different methods (B_low and C_only) at three different VAF cutoffs, 1%, 2.5%, and 5%. C_only was not applied to TFS as sample C was not tested on TFS. FP rates are plotted in squared root scale. **b** Estimated FP rate within the CTR averaged over three methods at different VAF cutoffs. **c** Cross-lab reproducibility (in Phred scale) in samples A and C within and outside of the CTR

Using the last two methods (“B_low” and “C_only”), we compared the FP rate within and outside of the CTR (Fig. 4a). By either method, across VAF ranges and across panels, we observed that the FP rate was lower in the CTR than outside. Breaking each panel into three sub-regions (inside the CTR, outside the CTR but in HC_CR, or the rest with the subregion sizes listed in Additional file 3: Fig. S3E), the FP rate was the lowest in the CTR and the highest outside of the HC_CR when using either the B_low or C_only method (Additional file 3: Fig. S3F-G). FP rates inside the CTR across different VAF cutoffs are listed in Fig. 4b. For all panels, the FP rate decreased as the VAF increased.

Finally, to better understand the impact of genomic regions on cross-lab reproducibility, we assessed cross-lab reproducibility within and outside of the CTR with VAF between 2.5 and 20% (Fig. 4c). Across most panels and in both samples A and C, cross-lab reproducibility was marginally lower outside of the CTR. Taken together, these results suggest that variant location may drive high cross-lab reproducibility seen in Fig. 2c. Since sensitivity did not drop noticeably outside the CTR (Fig. 3c), the observed drops in reproducibility (Fig. 4c) may be driven by more FPs outside of the CTR (Fig. 4a).

### Enabling oncopanels of sufficient size to estimate TMB

**Fig. 5:**
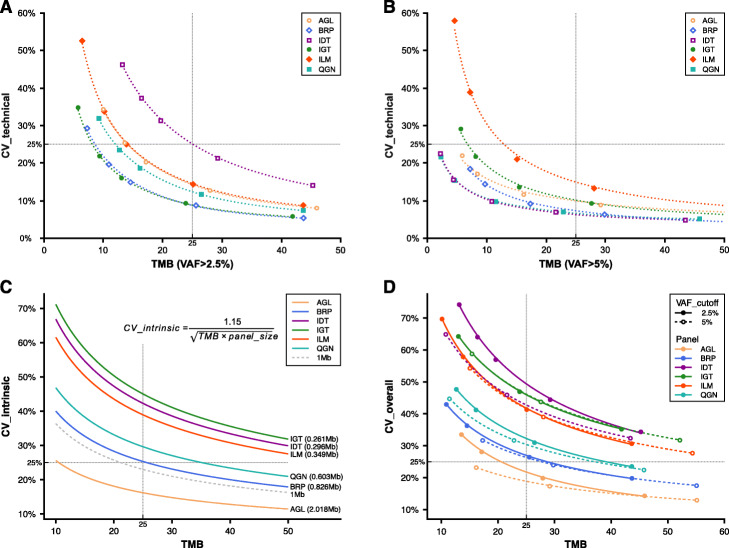
Coefficient of variation (CV) of TMB. **a** Technical run-to-run variance of TMB with VAF cutoff above 2.5% was estimated for six panels at different TMB levels. A power-law curve (dashed line) is fitted for each panel. **b** Technical run-to-run variance of TMB with VAF cutoff above 5%. **c** The intrinsic CV is plotted with the equation (embedded, see “Methods” for detail) for each panel based on their panel size. The curve (dashed) for size of 1 Mb is also plotted as a reference. **d** The overall CV is plotted combining technical and intrinsic CV. The solid fitting power-law curve is for TMB (VAF > 2.5%), and the dashed curve is for TMB (VAF > 5%)

Commonly measured by whole-exome sequencing (WES), TMB ranges over several orders of magnitude and varies by tumor type. Suitable TMB biomarker thresholds can also vary by tumor type, implying different degrees of accuracy may be required for different indications. The elevated FP rate outside of the CTR will have a strong detrimental effect on the accuracy of variant calls and subsequently TMB estimates. This leads to a reasonable requirement that TMB estimation should be confined to the CTR portion of each panel. The high density of variants in these reference samples was purposely designed to enable an accurate evaluation of sensitivity and limit of detection. To investigate a more “real-world” scenario where using an oncopanel to estimate exome-level TMB might be relevant, we randomly selected known variants comprised of a prevalence more consistent with actual tumors for a variety of indications (e.g., between 5 and 50 per Mb). We then computed the average technical run-to-run variance of panel-focused TMB estimates and plotted the technical coefficient of variation (CV) at two different VAF cutoffs: 2.5% (Fig. 5a) and 5% (Fig. 5b, see “Methods” for details). All six panels with targeted regions in the CTR greater than 250 kb were included in the analysis. Clearly, the technical CV decreased at higher panel TMB values. There was no observed effect related to the panel size. All panels achieved a CV lower than 25% when panel-focused TMB was 25 or higher.

To assess the magnitude of additional variance introduced by the genomic coverage of oncopanels, we utilized The Cancer Genome Atlas (TCGA) WES mutation data [36] to compute TMB estimates using each panel’s CTR portion. We then used the mean squared deviation (MSD) to measure the TMB estimation difference between whole-exome and the panel-specific CTR portion. MSD has two components: mean bias and variance. We characterized an intrinsic CV measure specific to each panel, related to this variance component. Further linear regression analysis led to an approximate equation estimating the intrinsic CV by TMB and the panel size (Additional file 3: Fig. S5, see “Methods” for details). The intrinsic CV is plotted in Fig. 5c. In agreement with technical CV, intrinsic CV decreases at higher TMB values. For example, a 1-Mb oncopanel is estimated to have an intrinsic CV of 25% at a TMB value of 21. By incorporating both technical variance and intrinsic variance, an overall CV for TMB was computed and plotted in Fig. 5d when using VAF cutoffs of 2.5% and 5% for each panel. Overall CV was close to intrinsic CV for each panel at corresponding TMB values. This indicates that all panels were able to achieve a lower technical variance than the intrinsic variance associated with estimating exome-level TMB from a smaller focused panel. Nevertheless, lower technical run-to-run variance is possible for all panels using a higher VAF cutoff as illustrated in Fig. 5d. This improvement was more apparent for AGL and QGN panels at lower TMB, and consistently large for IDT across a wide TMB range. Although many of the panels, e.g., QGN, were not designed for TMB estimation, they may be used to do so under certain circumstances. However, if the goal is to maintain an overall CV lower than 25% in the clinically important TMB range between 15 and 30, AGL combined with an VAF cutoff at 5% would be the only panel from these eight suitable for TMB estimation.

## Discussion

This oncopanel study was comprehensive in several areas: (1) the use of rigorous performance metrics both within and outside of easier/difficult genomic regions, (2) utilization of various oncopanel vendors with different panel sizes, (3) broad multi-laboratory participation, (4) analysis of a wide dynamic range of VAFs, and (5) use of test samples comprising advanced reference material with dense, known variant content from a stable source. We assessed reproducibility, sensitivity, and the FP rate of eight pan-cancer oncopanels across laboratories. We examined the impact on these metrics by VAF ranges, variant types, and genomic regions. These panels ranged in size from as small as 149 up to 7625 kb, and DNA input ranged from 20 to 100 ng. Enrichment of targets by both hybridization-based capture and PCR amplification were represented. The panel vendors represented major providers of currently marketed targeted sequencing technologies.

We cannot be completely certain that all of our results would generalize to a new admixture reference sample similarly constructed or real-world clinical formalin-fixed paraffin-embedded (FFPE) samples. Our primary reference sample (sample A) does not reflect all cancer indications, nor does it reflect all subtypes for any given cancer [26]. However, this approach provided noticeably better estimates than previous efforts [24]. For example, the large number of variants present in diverse sequence contexts in samples A and C enabled an accurate assessment of sensitivity and reproducibility for detecting SNVs. We observed that these panels detected known small indel or MNV positives with similar sensitivity and reproducibility as SNVs (Fig. 3a, Additional file 3: Fig. S2). However, with the limited amount of small indel/MNV positives identified as a proportion of all identified variants in the reference sample (1.2%), it is likely given prior research in the field that these small indel/MNV positives identified in the reference sample are in group that is easier to detect, in general. Therefore, we cannot suggest that these panels find small indels/MNVs with the same proficiency as SNVs throughout their target regions until we have identified a larger and more representative set of indels/MNVs from which to compare. While SNVs predominate clinically relevant oncology mutations, non-SNVs can also be key drivers [37], and panels should be further characterized for analysis of non-SNVs as these variants are typically more challenging to detect than SNVs. Additional reference material with more complex variants is required to address this outstanding question.

We chose to use the output of vendor-implemented bioinformatics pipelines for their respective proprietary panels. Other SEQC2 work has clearly demonstrated that choices of bioinformatics pipelines will influence the variant calling results in WES and whole-genome sequencing (WGS) [31]. Our raw data are made public to enable further evaluation of various bioinformatics pipelines by the community. This study did not characterize the effect of read coverage, which can be critical for low VAF variants. Low read coverage can reduce sensitivity and sometimes inflate the FP rate [38]. In addition, this study did not explore the impact of sample quality on variant calling. In the clinical setting, it can take considerable time before samples are processed, and samples are often subject to formalin fixation. A separate team from the Oncopanel Sequencing Working Group revealed the impact of formalin fixiation time and within block position on the performance of oncopanel sequencing [39]. Taken together, the experience gained from this study suggests that future analysis should focus on assessing the impact of bioinformatics pipelines, including variant callers, on sensitivity and FP rates.

The FP rate impacts TMB and overall confidence in the panel. To the best of our knowledge, this is the first study with a comprehensive analysis of the FP rate for oncopanels. Importantly, our results revealed that increasing the VAF reporting threshold reduced the FP rate across all VAF values within the CTR, but this effect was far less dramatic outside of the CTR. Therefore, one straightforward way to control the FP rate, at least within the CTR, is to adjust the VAF reporting threshold, which for different panel implementation modalities, may be optimally and more simply determined by assaying our reference samples and additional negative (normal) reference material using multiple technical replicates and cross-lab testing. On the contrary, if the goal is to have sensitive detection of clinically actionable mutations, where the FP rate over a region is not so relevant, lower variant-specific VAF threshold can be used without compromising accuracy through a novel synthetic spike-in control that directly measures the variant-specific and experiment-specific background errors. The control is presented in an accompanying SEQC2 manuscript [40].

To understand the impact of genomic region on sensitivity, reproducibility, and FP rates, we assessed all variants compared to those only within the CTR. Sensitivity was consistent for each panel between the CTR and HC_CR (Fig. 3c). However, because known variants were only available within HC_CR, we were not able to assess sensitivity for the regions outside of HC_CR. We hypothesize that sensitivity would be the same outside of HC_CR or slightly lowered. As for the FP rate, we observed multiple fold increases for all panels outside of the CTR when studying the impact of genomic region (Fig. 4a). Reproducibility was lower outside of the CTR (Fig. 4c). A plausible inference would be that the much higher FP rate outside of the CTR led to lower reproducibility in that region. “Real-world” clinical samples likely harbor much fewer low VAF variants (i.e., VAF < 20%) than sample A or C that was used here. With fewer variants, the effect of FP rate on reproducibility will be even more pronounced, i.e., the change in reproducibility will be greater between the CTR and outside of the CTR. In the extreme case of samples without any low VAF variants, e.g., a pure sample from a healthy infant, reproducibility will be completely dependent on the FP calls with no relationship to sensitivity. When reproducibility would play an important role in the specific application of oncopanel sequencing, for example, profiling multiple samples to study intratumor genetic heterogeneity, one may consider restricting the analysis to the CTR. Although most pathogenic mutations are within the CTR [26], restricting the analysis of clinical samples to the CTR may lead to the exclusion of certain mutations of interest. Currently, the CTR has excluded difficult regions and places likely to cause systematic sequencing errors. Extending the CTR will be a good direction for future work.

We noted that cross-lab reproducibility is similar to, but generally lower than intra-lab reproducibility (Additional file 3: Fig. S1A). The VAF range has a greater impact than the lab on reproducibility; however, given the increased variability across labs, we encourage cross-lab testing of oncopanels. If this is not possible, an alternative is more extensive testing within the same lab with different operators over an extended period. Furthermore, routine proficiency testing will help safeguard the performance, even within the same lab.

When screening clinically actionable mutations, FP variants are typically filtered by various processes, including variant prioritization (where only selected variants are considered for determining targeted therapy), and therefore have a small impact in final clinical decisions [10, 25]. In contrast, FP calls could easily confound TMB calculations and create a noninformative biomarker. Based on the findings of this large-scale cross-panel study, we propose different filtering and threshold-related methods for different modalities: variants for prioritization and targeted therapy selection may employ lower VAF reporting thresholds to achieve maximal sensitivity, while variants used in TMB calculations for immunotherapy purposes should consider higher VAF reporting thresholds or use VAF cutoffs commensurate with a low FP rate. Within this study, we have evaluated the impact of analytical variables on the detection of mutations and TMB estimation without reference to specific cancer subtypes. While the described relationships between analytical variables and TMB estimation are likely to remain constant regardless of cancer subtypes, the cancer type may inform the selection of oncopanel and VAF threshold to optimize diagnostic yields. For example, a greater mutation density, which is typical in lung cancers and melanomas, will enable a more confident estimation of TMB with lower overall CVs (as shown in Fig. 5d for high TMB values). Therefore, a moderately sized oncopanel with a higher VAF threshold may be sufficient. Alternatively, lower VAFs, which confound confident diagnosis, are typical in cancers with lower sample purity or greater intratumor heterogeneity, such as pancreatic cancers. In these cancers, a larger oncopanel with a lower VAF threshold may be required to confidently estimate TMB.

In summary, all eight panels achieved high sensitivity (ranging from 84.2 to 100%) for variants of low VAF between 1 and 5% (Fig. 2a). Applying an increasingly strict VAF cutoff reduces the FP rate, but also reduces sensitivity (Fig. 3b). VAF reporting thresholds create boundary effects due to stochastic sampling, where a variant may have an observed VAF just above the threshold in one replicate and but below in another, causing a decrease in sensitivity and reproducibility. When comparing performance metrics in different genomic regions, sensitivity remains consistent; however, the FP rate is higher and consequentially reproducibility is lower outside of the CTR. These oncopanels demonstrated low relative technical variability in estimating TMB; however, due to the greater variability in sampling a much smaller region of the exome (Fig. 5), our results suggest that, at a minimum, oncopanels will require 1 Mb in the CTR region to properly estimate TMB at levels commonly used for decision thresholds and may require even larger panels if TMB thresholds are needed below 10–15 mutations per megabase. Results are generally consistent with previous in silico studies [41–43]. This comprehensive study provides actionable guidelines for oncopanel sequencing and clear evidence that supports a simplified approach to assess the analytical performance of oncopanels. It will facilitate the rapid implementation, validation, and quality control of oncopanels in clinical use.

## Methods

### Testing samples

Realizing that there is no adequate reference genomic material to enable a transparent cross-lab study of oncopanels, the Oncopanel Sequencing Working Group developed and validated a genomic sample suitable for benchmarking oncopanel’s performance in detecting small variants of low allele frequency (AF). The design, validation process, and the results are reported in a companion paper [26]. This sample, termed “sample A,” is composed of an equal mass pooling of 10 gDNA samples prepared from Agilent’s Universal Human Reference RNA (UHRR) [34] cancer cell lines. Over 42K small variants are called with high confidence in the defined CTR of over 22 million bases. Briefly, CTR is the overlap of targeted regions of four whole-exome sequencing panels, UCSC coding genes, Ensemble exons, and NIST high-confidence genomic regions, followed by a removal of low complexity regions [26]. The majority of these small variants have a VAF below 20%, making sample A uniquely suitable for assessing the analytical performance of oncology panels. More specifically, about 7% of them are in variant allele frequency (VAF) range (1%, 2.5%), 18% in (2.5%, 5%), 25% in (5%, 10%), and 18% in (10%, 20%). Sample B is a gDNA sample from a normal male cell line (Agilent Human Reference DNA, Male, Agilent part #: 5190-8848). Sample C is a 1:1 mix of sample A and sample B. Sample C was included to increase the number of known variants with VAF between 1 and 2.5%. The spike-in sample is sample B with 5% AcroMetrix^29^ Spike hotspot synthetic controls (Thermo Fisher Scientific, Fremont, CA). Samples A, B, and C were prepared by Agilent and provided without charge to SEQC2 for study. The spike-in sample was prepared at Thermo Fisher Scientific (Fremont, CA) after receiving sample B from Agilent under their material transfer agreement. All four samples were stored in low-EDTA TE buffer (10 mM Tris, 0.1 mM EDTA, pH 8.0) at 20 ng/μl concentration.

### Participating panels, test sites, and study protocol

Study participation of oncopanel vendors was actively sought out through all available venues. A study plan was discussed in detail with each interested contact, emphasizing the cross-lab study design, transparency, and the requirement for eventual data release to the public domain. Eight panel providers agreed to participate. Each panel provider then recruited independent test sites (i.e., laboratories) that were proficient with its panel and oncology sequencing procedure. Test samples were distributed to test sites after executing Agilent material transfer agreement and SEQC2 confidentiality agreement. A sample processing and sequence data reporting standard operating procedure (SOP) was also distributed to all test sites. It includes test sample description, sample receiving and preparation, a list of test sites, naming convention, testing procedure, and instructions on library preparation quality data collection. There were at least three independent test sites for each participating panel. Panel providers and their supporting partners distributed panels and reagents to the corresponding test sites. Each site followed the SOP and a panel-specific experimental protocol (see Additional file 2: Supplementary Methods for details) to make 4 replicate libraries for each sample. Sequencing was done on the same sequencing platform for each panel. Each panel provider determined the depth of coverage per library and sample C libraries were sequenced twice as deep as other samples. Panel providers were encouraged to conduct pilot sequencing experiments with test samples at their internal lab or test sites. Pilot data was not required for submission to SEQC2.

### Reproducibility by VAF ranges per panel

Given the variability in VAF measurements and its influence on reproducibility, a non-symmetric reproducibility measure was devised. For comparing library “*Libx*” to another replicate “*Liby*” of the same sample, reproducibility is defined as the portion of *Libx*’s variant calls that are also detected in *Liby*. It can be computed by dividing the number of variants called in both *Libx* and *Liby* by the number of variant calls in *Libx*. Similarly, a reproducibility value can be computed for comparing *Liby* to *Libx*. These two reproducibility values are not symmetric and thus may be different. For comparing *Libx* to *Liby*, VAF ranges are applied unsymmetrically to compute reproducibility through stratifying variant calls of *Libx* into VAF ranges with no restriction imposed to variant calls of *Liby*. When both library replicates are from a same test lab, it is counted as one intra-lab reproducibility measurement. When they are from different test labs, it is counted as one cross-lab reproducibility measurement. As there are multiple library replicates from multiple labs, many reproducibility measurements can be computed for each sample. The average intra-lab reproducibility indicates what percentage of variants called by one library in one lab within a specific VAF range will be detected by another library replicate in the same lab without any restriction to their VAF values. The average cross-lab reproducibility may be interpreted similarly.

### Sensitivity estimation by VAF ranges

Sensitivity is defined as the portion of known variants detected by each panel sequencing experiment among all the KPs targeted by the panel. As the list of KPs was not all inclusive, sensitivity was provided as an estimate. Fortunately, the number of known variants was usually high enough to provide an accurate estimate. For each sensitivity estimate, a 95% confidence interval was calculated by bootstrap resampling with the assumption that library replicates are not independent. Sensitivity estimates can be further stratified by the VAF of known variants. Four VAF ranges were adopted in this study, 1–2.5%, 2.5–5%, 5–10%, and 10–20%. For the spike-in sample, there was a single range around 5% as all AcroMetrix variants were spiked-in at a VAF of 5%.

### FP rate estimation approaches

Three approaches were adopted to estimate false positive (FP) rate. In the CTR, a list of known negative (KN) positions was provided for sample A. This list covers over 50% of the CTR, enough for an accurate estimation of FP rate. By removing the positions overlapping with any known variants in sample B, a separate list of KN positions was generated for sample C. Any variant calls overlapping with the negative positions are flagged as FPs. FP rate is estimated as the ratio of FP calls out of every million KN positions. This is the first approach (coded as “viaKN”), applicable to sample A and sample C, but limited to the CTR.

By the second approach (coded as “B_low”), any calls in the normal sample B with VAF between 1 and 10% are marked as false positives (FPs). A pure diploid normal sample has variants at either 50% (heterozygous) or 100% (homozygous). Stochastic sampling and the influence of copy number variation lead to VAF deviation from those two values. As no extremely high copy number variation was observed in sample B, it is reasonable to assume that variant calls in sample B with VAF below 10% are FPs. Variant calls with VAF greater than 10% in any of other three sample B libraries at the same test sites will be excluded. The third approach (coded as “C_only”) leveraged sample mixture to differentiate FPs. Any variant in sample C must come from either sample A or sample B. If a variant is called in one sample C library, but not in any of four sample B libraries with VAF above 10% or any of four sample A libraries at the same test laboratory, it is an FP. This approach is more burdensome as all three samples and multiple libraries must be considered, but it does not rely on the assumption of sample B’s variant VAF range or the KNs in sample A. Furthermore, it cannot detect FPs due to any panel bias. Unlike “viaKN,” both “B_low” and “C_only” can be applied to estimate FP rate in the regions beyond the CTR.

### Description of three sub-regions for each panel

The CTR was defined as the overlapping regions of four whole-exome sequencing panels that were used in the companion work^21^ to characterize known variants in sample A, NIST high-confidence regions, and exome coding regions. The CTR was then refined by removing the low complexity regions. The CTR is completely within high-confidence exome coding regions (HC_CR) that is defined as the overlapping regions of NIST high-confidence regions and exome coding regions. By intersecting the CTR and HC_CR with the targeting region of each panel, three sub-regions for each panel can be defined for performance comparison: within the CTR, within the HC_CR but outside of the CTR, and the rest.

### Evaluation of technical run-to-run variability in panel TMB estimates via simulation

The simulation aimed to reduce the variant density and reduce low VAF variants to better mimic a “real-world” clinical sample. While the high variant density and high percentage of variants with VAF below 5% in the test samples provided an advantage in estimating detection sensitivity with narrow confidence intervals, they presented obstacles here for the evaluation of TMB technical variability. The high variant density (equivalent to a TMB value about 1000) in the test samples will overshadow the effect of the FP calls and thus greatly underestimate TMB technical variability. The high percentage (over 75% in sample C) of variants with VAF below 5% may lead to an overestimate of TMB technical variability due to the variability in detecting these low VAF variants. The simulation was applied to the CTR. The resultant median VAF for low-frequency (< 20%) KPs would be between 5 and 10%. Sample C was chosen for the simulation analysis as there are fewer uncharacterized true variants in sample C with VAF greater than 2.5% in comparison to sample A. All the known variants in sample B were excluded first from our KP list and from all the variant calling results (VCF files of library replicates) of each panel. The KPs with VAF < 5% or VAF > 40% in sample A were also removed. In theory, no KP with VAF < 2.5% or VAF > 20% in sample C was left. To mimic multiple samples with the constant TMB at a similar level with a clinical sample, we kept a fixed portion of KPs that resulted in the same number of KPs across the CTR for panel testing in the simulated samples. Specifically, based on their VAF in sample A, *k*% (*k* = 0, 1, 2, 5, 10, 20) of KPs were retained for VAF between 10 to 40% and 0.5 × *k*% of KPs were kept for VAF between 5 and 10%. Given each value, (100 − *k*)% of KPs were randomly excluded from CTR to generate a base-report-region for all the pan-cancer panels. Therefore, the variants not excluded from a VCF file were counted as the reported calls by the library of a pan-cancer panel in a simulation run. It is worth pointing out that the reported variants include FP calls and possibly uncharacterized true variants in additional to the retained KPs. Finally, 5000 rounds of simulation were performed for each based-report-region with a *k* value greater than 0.

For each one of 5000 base-report-region with a *k* value (*k* > 0), the reported variants were then counted for these above a chosen VAF threshold for each library replicate. The variance and mean across all technical replicates for each panel were calculated. Then, we computed the overall average of variance and mean over the 5000 runs of simulation. Finally, the CV for TMB were calculated as:

$$ \mathrm{CV}=\frac{\sqrt{\mathrm{Ovarall}\ \mathrm{Average}\ \mathrm{of}\ \mathrm{Variance}}}{\mathrm{Ovarall}\ \mathrm{Average}\ \mathrm{of}\ \mathrm{Mean}\ \mathrm{Count}} $$

The mean TMB for each chosen *k* value was calculated as the overall average count of variants divided by the report region sizes of six panels. This CV is adopted to measure the technical run-to-run variability and examine its dependence on TMB and VAF cutoff.

### Model the deviation in TMB rate from WES by pan-cancer panels via TCGA dataset

Mutation Annotation Format (MAF) files of 10,295 cancer genomes across 33 cancer types were collected from The Cancer Genome Atlas (TCGA) project involving 2.9 million somatic mutations (MC3 Public MAF) for TMB measurement. The shared portion of TCGA coding sequence (CDS) region and the CTR with a size of 22,089,460 bases (55% of TCGA CDS) was used to calculate the TMB rate of each individual cancer sample. The TCGA-measured TMB rate was calculated as the number of mutations per million bases within the shared region and used as the baseline in this analysis. Then, given the TCGA somatic mutations as ground truth, the panel-measured TMB rate of each cancer sample was calculated by the number of mutations within the panel divided by the panel’s region overlapping with the CTR and TCGA CDS in million base pairs. Two panels, ROC and TFS, were excluded from this analysis because of their small sizes.

For each pan-cancer panel, the mean squared deviation (MSD) was utilized to evaluate the difference between TCGA and panel TMB rates of the same group of cancer samples,

$$ \mathrm{MSD}\left({P}_j\right)=\frac{\sum_{i=1}^n{\left(\mathrm{TCGA}.{\mathrm{TMB}}_i-{P}_j\left(i,{\mathrm{TMB}}_i,{\mathrm{Cov}}_j\right)\right)}^2}{N} $$

where *j* represents each panel and *i* is an individual cancer sample. The function *P*_*j*_ calculates the TMB of sample *i* within the covered region (Cov_*j*_) by panel *j*. *N* represents the number of samples in the group for the calculation of MSD. One outlier cancer genome TCGA-13-0889-01A-01W-0420-08 (with TCGA TMB rate of 33) was excluded from the calculation of MSD due to the abnormally huge difference between the TMB rates measured by TCGA WES and panels.

For a group of samples with TMB rates close to each other and thus close to their mean value, a modified MSD (MSD´) is defined by substituting the TMB mean for the individual TCGA TMB in the equation above. Approximately, MSD equals to MSD´ subtracting the variance of TCGA TMB in the sample group. Mathematically, MSD´ equals to the sum of the variance of TMB by the panel and the squared difference in the mean TMB values between TCGA and the panel. Thus, MSD is broken down into two components of mean bias and variance (see Additional file 2: Supplementary Methods for details). The mean bias component is equal to the squared difference in the mean TMB values between TCGA and the panel. The variance component is equal to the variance of TMB by the panel reduced by the variance of TCGA TMB. This adjusted variance component is of particularly interest.

To better understand this adjusted panel TMB variance, we selected the TCGA samples (about 2400 samples) with TMB rates from 5 to 40 and sorted them by TMB. We then grouped each 100 samples and calculated the panel TMB mean value and the adjusted panel TMB variance. Multiplying it by the squared panel size in million bases, we converted the adjusted panel TMB variance to the mutation count level, which was then plotted against the product of the panel TMB mean and the panel size. A linear regression model was fitted over these two variables from all the six panels that cover at least 250 kb in the CTR (Additional file 3: Fig. S5). A very high *R*^2^ value (0.957) was observed along with a tight 95% confidence interval (1.28–1.34) for the slope of the regression line. This revealed a strong linear relationship between the adjust panel TMB variance and the panel TMB mean in mutation count. Finally, an intrinsic CV for each panel could be calculated as 1.15 divided by the square root of panel TMB mean in mutation count.

## Supplementary Information

**Additional file 1: Table S1.** Detailed information for eight participating pan-cancer panels. **Table S2.** 95% confidence interval for reported sensitivity across VAF ranges for SNVs in the consensus targeted region (CTR). **Table S3.** 95% confidence interval for reported sensitivity in detecting known SNVs and other variants of expected VAF between 2.5% and 20%. **Table S4.** 95% confidence interval for reported sensitivity in detecting known SNVs within the CTR of expected VAF between 2.5% and 5% after applying the artificial VAF cutoff at 1.5%, 2.0% and 2.5%. **Table S5.** Sensitivity across VAF ranges for all samples after applying the artificial VAF cutoff. **Table S6.** 95% confidence interval for reported sensitivity within the CTR or HC_CR (more specifically, in HC_CR beyond the CTR) in detecting known positives of expected VAF between 2.5% and 20%.

**Additional file 2.** Supplementary Methods.

**Additional file 3: Fig. S1.** Concordance of average cross-lab reproducibility and intra-lab reproducibility in Phred scale across VAF ranges for samples A and C. **Fig. S2.** Comparison of sensitivity of different variant types across VAF ranges. **Fig. S3.** Estimation of the false positive rate by three methods. **Fig. S4.** Low reproducibility of false positive calls across library replicates. **Fig. S5.** Scatter plot of panel TMB mean × panel_size (x-axis) and adjusted panel TMB variance × panel_size2 (y-axis).

**Additional file 4.** Review history.

## Data Availability

Important data items from the consortium oncopanel sequencing effort that we intend to disclose include the following: (1) NGS sequencing data [44] (in FASTQ or BAM format) from eight oncopanels for sample A, sample B, sample C, and the spike-in sample; (2) variant call results [45] (in VCF format) by the panel providers for these four samples; and (3) dependent files [45] (e.g., panel BED files, genome FASTA files) for each oncopanel.
