# Supplementary material for: Cross-oncopanel study reveals high sensitivity and accuracy with overall analytical performance depending on genomic regions
Source: Genome Biol. 2021 Apr 16;22:109. doi: 10.1186/s13059-021-02315-0 (PMC8051090; doi:10.1186/s13059-021-02315-0)
Supplement: Supplementary file 1 — Additional file 1: Table S1. Detailed information for eight participating pan-cancer panels. Table S2. 95% confidence interval for reported sensitivity across VAF ranges for SNVs in the consensus targeted region (CTR). Table S3. 95% confidence interval for reported sensitivity in detecting known SNVs and other variants of expected VAF between 2.5% and 20%. Table S4. 95% confidence interval for reported sensitivity in detecting known SNVs within the CTR of expected VAF between 2.5% and 5% after applying the artificial VAF cutoff at 1.5%, 2.0% and 2.5%. Table S5. Sensitivity across VAF ranges for all samples after applying the artificial VAF cutoff. Table S6. 95% confidence interval for reported sensitivity within the CTR or HC_CR (more specifically, in HC_CR beyond the CTR) in detecting known positives of expected VAF between 2.5% and 20%. [file 13059_2021_2315_MOESM1_ESM.pdf]

Table S1: Detailed information for eight participating pan-cancer panels

Panel code	Panel name*	List of test labs	Size (Kbp)	Gene count	Genome version	DNA input (ng)
AGL	Agilent Custom Comprehensive Cancer Panel v2	ST01/ST02/ST03	7,625	1058	hg19	30
BRP	Burning Rock DX OncoScreen Plus	ST25†/ST26/ST27/ST28	1,631	523	hg19	100
IDT	Integrated DNA Technologies xGen Pan-Cancer Panel	ST04/ST05/ST06	780	127	hg19	100
IGT	iGeneTech AIOnco-seq	ST07/ST08/ST09	944	113	hg19	100
ILM	Illumina TruSight Tumor 170	ST10/ST11‡/ST12/ST23/ST29	527	154	hg19	50
QGN	QIAGEN Human Comprehensive cancer panel	ST13/ST14/ST15	837	275	hg19	40
ROC	Roche SeqCap EZ Choice custom PHC Panel	ST16/ST17§/ST18/ST19	149	45	hg38	100
TFS	Thermo Fisher Oncomine Comprehensive Assay v3	ST22/ST23/ST24	349	146	hg19	20

(continue…)

Panel code	UMI	Fragmentation approach	Median fragment size (bp)
AGL	Yes (for deduplication)	Covaris E220 instrument / Focused-Ultrasonicators	350
BRP	No	Covaris M220/Focused-Ultrasonicators with AFA Technology	265
IDT	No	Covaris / Focused-Ultrasonicators	300
IGT	No	Covaris / Focused-Ultrasonicators	200 (150-250)
ILM	No	Covaris / Focused-Ultrasonicators	170 (90-250)
QGN	Yes (for error reduction)	QIAGEN Fx enzymatic fragmentation module	183
ROC	Yes (for deduplication)	Kapa Plus enzyme	187
TFS	No	No fragmentation as PCR based target amplification	156

(continue…)

Panel code	Enrichment	Sequencing platform	Read length	Avg. read count
AGL	capture based/Agilent SureSelectXT HS Target Enrichment System	NovaSeq	2 x 150bp	362,985,832
BRP	capture based	NovaSeq	2 x 150bp	89,735,363
IDT	capture based	NovaSeq	2 x 150bp	124,947,599
IGT	capture based	HiSeq2500	2 x 125bp	101,613,666
ILM	capture based	NextSeq	2 x 101bp	>100 million
QGN	one end randomly fragmented and adapter ligated, other end gene specific primer, single primer extension and universal PCR	NovaSeq	2 x 150bp	141,196,120
ROC	capture based	NovaSeq	2 x 150bp	422,956,489
TFS	amplicon based	IonTorrent S5	113 bp (average)	10,765,203

(continue…)

Panel code	Avg. raw read coverage	Avg. coverage after deduplication	Read mapping tool	Variant caller	VAF threshold
AGL	7,141	3,550	bwa mem v0.7.17	GATK 4.0.10.0 Mutect2	1%
BRP	4,682	2,194	BWA aligner 0.7.10	VarScan v2.4.3	1%
IDT	10,179	3,026	bwa-mem v0.7.15	AstraZeneca VarDict v1.4.8	2%
IGT	7,359	4,508	bwa mem v0.7.12	VarScan.v2.3.7	1%
ILM	9,880	1,412	iSAAC aligner	Pisces variant caller	2.6%
QGN	22,868	2,431	BWA-MEM	UMI-aware variant caller smCounter2	No‖
ROC	>30,000	5,767	BWA 0.7.17	GATK 4.1.0.0 Mutect2	2.5%
TFS	3,191	We do not de duplicate bams for variant calling rather TVC maxes out at read depth of 2000x	TMAP	Torrent Variant Caller (TVC)	2.5%

*All participating panels are for research use only.
† ST25 was excluded from performance analysis as it is a clinical lab closely affiliated with the panel provider.
‡ ST11 was excluded from performance analysis due to an extended delay in experiment execution and data generation.
§ ST17 was excluded from performance analysis due to over fragmentation of DNA samples during library preparation.
‖ QGN's UMI-aware variant caller is able to call variants with VAF as low as 0.5%.

Table S2: 95% confidence interval for reported sensitivity across VAF ranges for SNVs in the consensus targeted region (CTR).

	Sample A				Sample C				Spike-in
VAF range	**1-2.5%**	**2.5-5%**	**5-10%**	**10-20%**	**1-2.5%**	**2.5-5%**	**5-10%**	**10-20%**	**~5%**
**AGL**	[84.4%, 89.6%]	[96.1%, 97.4]	[98.2%, 99.3%]	[99.9%, 100%]	[89.6%, 91.5%]	[97.6%, 98.9%]	[98.7%, 100%]	[98.6%, 100%]	[95.9%, 98.4%]
**BRP**	[91.2%, 97.4%]	[99.0%, 100%]	[99.9%, 100%]	*	[93.4%, 96.1%]	[99.8%, 100%]	*	*	[99.9%, 100%]
**IDT†**	N/A	[97.2%, 99.5%]	*	*	N/A	[96.7%, 99.5%]	*	*	[96.9%, 98.9%]
**IGT**	[92.4%, 100%]	*	*	*	[97.3%, 99.0%]	*	*	*	*
**ILM†**	N/A	[85.6%, 91.8%]	[96.5%, 99.7%]	[99.5%, 100%]	N/A	[80.1%, 98.2%]	[99.4%, 100%]	[90.1%, 100%]	[92.8%, 95.9%]
**QGN**	[91.4%, 97.6%]	[99.0%, 99.8%]	[99.0%, 100%]	*	[94.3%, 96.6%]	[99.1%, 100%]	*	[98.1%, 100%]	[92.2%, 96.4%]
**ROC†**	N/A	[80.7%, 95.5%]	*	*	N/A	[69.2%, 94.3%]	*	*	[84.4%, 91.3%]
**TFS†**	N/A	[88.8%, 95.8%]	[99.5%, 100%]	*	NOT TESTED				[99.5%, 100%]

* The estimate for sensitivity is 100% across all libraries thus bootstrap resampling is not applicable for calculating the confidence interval.
† For the panels with a built-in VAF threshold, "N/A" is listed if the VAF range's low bound is much lower than the panel provider's chosen VAF threshold. The VAF threshold is 2.0% for IDT, 2.6% for ILM, 2.5% for ROC, and 2.5% for TFS.

Table S3: 95% confidence interval for reported sensitivity in detecting known SNVs and other variants of expected VAF between 2.5% and 20%.

	Sample A		Sample C	
Variant type	**SNV**	**Small indels or MNVSs**	**SNV**	**Small indels or MNVSs**
**AGL**	[97.5%, 98.3%]	[88.2%, 98.6%]	[98.3%, 99.3%]	[87.3%, 100%]
**BRP**	[99.4%, 99.9%]	*	[99.7%, 100%]	*
**IDT**	[98.5%, 99.8%]	*	[98.3%, 99.6%]	*
**IGT**	*	*	*	*
**ILM**	[92.0%, 95.4%]	[94.3%, 99.0%]	[88.6%, 94.0%]	[48.4%, 100%]
**QGN**	[99.3%, 99.8%]	*	[99.3%, 99.9%]	*
**ROC**	[91.1%, 98.1%]	*	[87.0%, 97.8%]	[25%, 100%]

* The estimate for sensitivity is 100% across all libraries thus bootstrap resampling is not applicable for calculating the confidence interval.

Table S4: 95% confidence interval for reported sensitivity in detecting known SNVs within the CTR of expected VAF between 2.5% and 5% after applying the artificially VAF cutoff at 1.5%, 2.0% and 2.5%.

	Sample A				Sample C			
Artificially applying VAF cutoff	**>2.5%**	**>2%**	**>1.5%**	**>1%**	**>2.5%**	**>2%**	**>1.5%**	**>1%**
**AGL**	[88.4%, 90.5%]	[93.5%, 95.1%]	[95.5%, 96.9%]	[96.0%, 97.3%]	[84.5%, 87.9%]	[92.7%, 94.8%]	[96.5%, 98.0%]	[97.3%, 98.8%]
**BRP**	[88.2%, 92.1%]	[96.1%, 98.1%]	[98.5%, 99.6%]	[99.0%, 99.9%]	[85.5%, 91.0%]	[95.0%, 97.6%]	[99.1%, 99.8%]	[99.5%, 100%]
**IDT†**	[93.4%, 97.9%]	[97.3%, 99.6%]	N/A	N/A	[83.8%, 94.2%]	[97.0%, 99.4%]	N/A	N/A
**IGT**	[93.2%, 97.9%]	[98.7%, 99.9%]	*	*	[91.9%, 97.6%]	[99.7%, 100%]	*	*
**QGN**	[93.3%, 96.6%]	[97.5%, 99.0%]	[98.8%, 100%]	[99.0%, 99.8%]	[86.6%, 92.7%]	[95.9%, 98.5%]	[98.7%, 99.8%]	[99.1%, 99.9%]

(continue...)

	Spike-in			
Artificially applying VAF cutoff	**>2.5%**	**>2%**	**>1.5%**	**>1%**
**AGL**	[92.9%, 95.6%]	[95.2%, 97.5%]	[95.9%, 98.2%]	[96.1%, 98.3%]
**BRP**	[96.6%, 98.6%]	[97.9%, 99.5%]	[98.4%, 99.8%]	[98.9%, 100%]
**IDT†**	[95.5%, 97.9%]	[97.0%, 98.8%]	N/A	N/A
**IGT**	[98.3%, 99.8%]	[98.8%, 100%]	[98.8%, 100%]	[99.0%, 100%]
**QGN**	[88.1%, 92.9%]	[89.6%, 94.2%]	[99.3%, 99.8%]	[90.8%, 95.1%]

* The estimate for sensitivity is 100% across all libraries thus bootstrap resampling is not applicable for calculating the confidence interval.
† "N/A" is listed in some VAF ranges for IDT if IDT's chosen VAF threshold, 2.0%, is much higher than the VAF range's low bound.

Table S5: Sensitivity across VAF ranges for all samples after applying the artificial VAF cutoff

a Sensitivity with the artificial VAF cutoff at 1.5%

	Sample A								Sample C								Spike-in	
KP AF range	**1-2.5%**		**2.5-5%**		**5-10%**		**10-20%**		**1-2.5%**		**2.5-5%**		**5-10%**		**10-20%**		**~5%**	
**AGL**	*207*	75.5%	*1,002*	96.3%	*628*	98.6%	*395*	99.8%	*1,102*	78.3%	*572*	97.3%	*300*	99.4%	*246*	99.6%	*483*	97.1%
**BRP**	*83*	77.3%	*431*	99.1%	*269*	99.9%	*170*	100%	*470*	80.8%	*251*	99.5%	*132*	100%	*99*	99.9%	*488*	99.2%
**IDT**	*31*	*NA*	*156*	95.8%	*88*	100%	*51*	100%	*175*	*NA*	*82*	89.4%	*40*	100%	*36*	99.7%	*246*	96.8%
**IGT**	*24*	90.3%	*140*	100%	*81*	100%	*53*	100%	*155*	86.8%	*77*	100%	*40*	100%	*33*	100%	*429*	99.5%
**ILM**	*40*	*NA*	*199*	88.8%	*113*	98.5%	*83*	99.9%	*222*	*NA*	*106*	84.6%	*63*	99.8%	*39*	95.8%	*477*	93.4%
**QGN**	*68*	81.7%	*317*	99.3%	*197*	99.5%	*104*	100%	*356*	83.7%	*184*	99.3%	*84*	100%	*73*	99.4%	*373*	92.6%
**ROC**	*17*	*NA*	*54*	89.5%	*36*	100%	*28*	100%	*66*	*NA*	*34*	83.6%	*23*	100%	*11*	100%	*335*	85.9%
**TFS**	*22*	*NA*	*125*	91.9%	*58*	99.7%	*42*	97.6%	*NOT TESTED*								*244*	98.7%

b Sensitivity with the artificial VAF cutoff at 2%

	Sample A								Sample C								Spike-in	
KP AF range	**1-2.5%**		**2.5-5%**		**5-10%**		**10-20%**		**1-2.5%**		**2.5-5%**		**5-10%**		**10-20%**		**~5%**	
**AGL**	*207*	55.9%	*1,002*	94.3%	*628*	98.6%	*395*	99.7%	*1,102*	55.2%	*572*	93.8%	*300*	99.4%	*246*	99.6%	*483*	96.4%
**BRP**	*83*	48.0%	*431*	97.2%	*269*	99.8%	*170*	100%	*470*	52.4%	*251*	96.4%	*132*	100%	*99*	99.9%	*488*	98.8%
**IDT**	*31*	*NA*	*156*	95.8%	*88*	100%	*51*	100%	*175*	*NA*	*82*	89.4%	*40*	100%	*36*	99.7%	*246*	96.8%
**IGT**	*24*	70.5%	*140*	99.4%	*81*	100%	*53*	100%	*155*	66.0%	*77*	99.9%	*40*	100%	*33*	100%	*429*	99.5%
**ILM**	*40*	*NA*	*199*	88.8%	*113*	98.5%	*83*	99.8%	*222*	*NA*	*106*	84.6%	*63*	99.8%	*39*	95.8%	*477*	93.4%
**QGN**	*68*	59.3%	*317*	98.3%	*197*	99.5%	*104*	100%	*356*	59.1%	*184*	97.3%	*84*	100%	*73*	99.4%	*373*	92.0%
**ROC**	*17*	*NA*	*54*	89.5%	*36*	100%	*28*	100%	*66*	*NA*	*34*	83.6%	*23*	100%	*11*	100%	*335*	85.9%
**TFS**	*22*	*NA*	*125*	91.9%	*58*	99.7%	*42*	97.6%	*NOT TESTED*								*244*	98.7%

c Sensitivity with the artificial VAF cutoff at 2.5%

	Sample A								Sample C								Spike-in	
KP AF range	**1-2.5%**		**2.5-5%**		**5-10%**		**10-20%**		**1-2.5%**		**2.5-5%**		**5-10%**		**10-20%**		**~5%**	
**AGL**	*207*	32.7%	*1,002*	89.5%	*628*	98.4%	*395*	99.7%	*1,102*	30.3%	*572*	86.2%	*300*	99.3%	*246*	99.6%	*483*	94.3%
**BRP**	*83*	19.2%	*431*	90.2%	*269*	99.8%	*170*	100%	*470*	22.4%	*251*	88.3%	*132*	100%	*99*	99.9%	*488*	97.7%
**IDT**	*31*	*NA*	*156*	95.8%	*88*	100%	*51*	100%	*175*	*NA*	*82*	89.4%	*40*	100%	*36*	99.7%	*246*	96.8%
**IGT**	*24*	23.3%	*140*	95.7%	*81*	100%	*53*	100%	*155*	25.2%	*77*	95.0%	*40*	100%	*33*	100%	*429*	99.1%
**ILM**	*40*	*NA*	*199*	88.8%	*113*	98.5%	*83*	99.8%	*222*	*NA*	*106*	84.6%	*63*	99.8%	*39*	95.8%	*477*	93.4%
**QGN**	*68*	23.8%	*317*	95.0%	*197*	99.5%	*104*	100%	*356*	23.9%	*184*	89.7%	*84*	100%	*73*	99.4%	*373*	90.6%
**ROC**	*17*	*NA*	*54*	89.5%	*36*	100%	*28*	100%	*66*	*NA*	*34*	83.6%	*23*	100%	*11*	100%	*335*	85.9%
**TFS**	*22*	*NA*	*125*	91.9%	*58*	99.7%	*42*	97.6%	*NOT TESTED*								*244*	98.7%

Table S6: 95% confidence interval for reported sensitivity within the CTR or HC_CR (more specifically, in HC_CR beyond the CTR) in detecting known positives of expected VAF between 2.5% and 20%.

	Sample A		Sample C	
Variant type	**within CTR**	**in HC_CR beyond CTR**	**within CTR**	**in HC_CR beyond CTR**
**AGL**	[97.6%, 98.3%]	[91.8%, 98.8%]	[98.3%, 99.3%]	[95.3%, 100%]
**BRP**	[99.5%, 100%]	[97.6%, 100%]	[99.9%, 100%]	[97.7%, 100%]
**IDT**	[98.5%, 99.8%]	*	[98.3%, 99.7%]	[96.8%, 100%]
**IGT**	*	*	*	*
**ILM**	[92.2%, 95.5%]	[81.6%, 100%]	[88.0%, 93.7%]	[86.8%, 100%]
**QGN**	[99.3%, 99.8%]	[98.7%, 100%]	[99.3%, 99.9%]	[99.0%, 100%]
**ROC**	[91.1%, 98.0%]	*	[83.0%, 96.6%]	*
**TFS**	[92.2%, 97.0%]	[97.9%, 100%]	NOT TESTED	

* The estimate for sensitivity is 100% across all libraries thus bootstrap resampling is not applicable for calculating the confidence interval.
