# Supplementary material for: Cross-oncopanel study reveals high sensitivity and accuracy with overall analytical performance depending on genomic regions
Source: Genome Biol. 2021 Apr 16;22:109. doi: 10.1186/s13059-021-02315-0 (PMC8051090; doi:10.1186/s13059-021-02315-0)
Supplement: Supplementary file 2 — Additional file 2. Supplementary Methods. [file 13059_2021_2315_MOESM2_ESM.pdf]

Cross-oncopanel study reveals high sensitivity and accuracy with overall analytical performance depending on genomic regions

SEQC2 Oncopanel Sequencing Working Group

Supplementary Methods

AGL experiment protocol

Genomic DNA libraries were constructed for the test samples according to the Agilent SureSelectXT HS Target Enrichment System for Illumina Paired-End Multiplexed Sequencing Library Protocol (Cat. No. G9702-90000 Version A1, July 2017). In brief, 30ng of each cell line high molecular weight genomic DNA was sonicated in a 50µl total volume with a Covaris E220 instrument to a mean size of 350bp (Duty Factor: 10%, Peak Incident Power: 175, Cycles per Burst: 200, Treatment Time: 2 × 30 seconds, Bath Temperature: 2° to 8°C). DNA fragments were then end-repaired and A-tailed using a two-step cycling protocol (20°C for 15 minutes and 72°C for 15 minutes), followed by ligation to XTHS adaptors with UMIs for 30 minutes at 20°C. Adapter-ligated fragments were amplified and indexed by PCR in a 50µl total volume with Herculase II Fusion DNA Polymerase under the following conditions: 2 min at 98°C (initial denaturation), 10 cycle amplification of 30 seconds at 98°C, 30 seconds at 60°C, 1 minute at 72°C, and 5 minutes at 72°C (final extension). Library quality control (quantity and size distribution) was then assessed using either the 2100 Bioanalyzer and DNA 1000 assay or the 2200 TapeStation and D1000 screen tape. 1µg of prepared gDNA libraries were then hybridized to a custom Immuno-Oncology focused Comprehensive Cancer Panel (1058 targets coding regions including UTRs and 7.6Mb in size) biotinylated RNA probes (5 minutes at 95°C, 10 minutes at 65°C, 1 minute at 65°C, 60 cycles of 1 minute at 65°C and 3 seconds at 37°C, and 65°C hold) and captured with Dynabeads MyOne Streptavidin T1 beads. SureSelect enriched gDNA libraries were PCR amplified using on-bead protocol in a 50µl total volume with Herculase II Fusion DNA Polymerase under the following conditions: 2 min at 98°C (initial denaturation), 10 cycles of 30 seconds at 98°C, 30 seconds at 60°C, 1 minute at 72°C (amplification), and 5 minutes at 72°C (final extension), followed by 4°C hold. All DNA purifications between steps were performed with AMPure XP beads as indicated in the user manual. Post-capture library quality control was assessed using either the 2100 Bioanalyzer and High Sensitivity DNA assay or the 2200 TapeStation and HSD1000 screen tape. Indexed samples were finally pooled and sequenced to approximately 5000X (Samples A, B, AC5, AAG) or 10,000X (Sample C) read depth on a NovaSeq 6000 instrument using a 2x150bp Paired-End protocol (Q30 scores ≥ 75%).

AGL bioinformatics pipeline

Each sample was demultiplexed using bcl2fastq[1] with the base mask Y150, I8, Y10, Y150 and all default settings except for mask-short-adapter-reads, which was set to 0. Adapters were trimmed using AGeNT Trimmer (Agilent)[2]. All data was aligned to the hg19 reference genome using bwa mem v1.7.17[3] with default settings. Quality control was performed using Picard tools[4] and an internally developed pipeline. Deduplication was performed using AGeNT LocatIt (Agilent)[2], allowing 2 mismatches between molecular barcodes and requiring at least two reads supporting each molecular barcode. Variant calls were done with GATK 4.0.10.0 Mutect2[5], after applying the GATK4 Base Recalibrator[6]. Mutect2 was run with all default settings, except max-readers-per-alignment-start was set to 0 and the read filter MatchingBasesAndQualsReadFilter was applied.

BRP experiment protocol

The library prep and enrichment process were performed using Burning Rock HS library preparation kit without modification. In brief, DNA shearing was performed on SEQC2 gDNA test samples using Covaris M220 for 195S, with Peak Incident Power=50W, Duty Factor: 20%, Cycle Per Burst: 200, at 6-8℃, followed by end repair, adaptor ligation and PCR enrichment. About 1µg of purified pre-enrichment library were hybridized to OncoScreenPlusTM panel and further enriched following manufacturer instruction. The OncoScreenPlusTM panel is about 1.7M bp in size and covers 520 human cancer related genes. Final DNA libraries were quantified using Qubit Fluorometer with dsDNA HS assay kit (Life Technologies, Carlsbad, CA). A LabChip GX Touch System, Agilent 2100 bioanalyzer or Agilent 4200 TapeStation D1000 ScreenTape was then performed to assess the quality and size distribution of the library. The libraries were sequenced on NovaSeq 6000 sequencer (Illumina, Inc., California, US) with 2×150bp pair-end reads with unique dual index.

BRP bioinformatics pipeline

After demultiplex using bcl2fastq v2.20 (Illumina)[1], sequence data were filtered using the Trimmomatic 0.36[7] with parameters "TRAILING:20 SLIDINGWINDOW:30:25 MINLEN:50". Sequence data were mapped to the human genome (hg19) using BWA aligner 0.7.10[3]. Local alignment optimization, variant calling and annotation were performed using GATK v3.2.2[6] with parameters "--interval_padding 100 -known 1000G_phase1.indels.b37.vcf -known Mills_and_1000G_gold_standard.indels.b37.vcf" and VarScan v2.4.3 with parameters "-min-coverage 50 --min-var-freq 0.005 --min-reads2 5 --output-vcf 1 --strand-filter 0 --variants 1 --p-value 0.2". For SNV and small indels, variants were further filtered using the homebrew variant filter pipeline. For each valid variant, the covered raw depth must be greater or equal than 50 (DP>=50), and at least 5 mutation supporting count (AD>=5), minor allele frequency needs to be greater than 0.01 (AF>=0.01). In order to further filter out false positives, only variant with at

least 6 unique fragments support or 2 unique paired fragments, i.e., within overlapping region between read pairs, support were kept. After filtering, remaining valid variants were annotated with ANNOVAR 20160201[8] and SnpEff v3.6[9]. For SNV and Indel, only the mutations located within coding exon and corresponding 20bp flanking region were reported in final VCFs.

IDT experiment protocol

Libraries were constructed using 100 ng each of test samples in quadruplicate. Briefly, genomic DNA was sheared to 300bp (Covaris) followed by library construction using the NEBNext Ultra II DNA Library Prep Kit for Illumina and xGen Dual Index UMI Adapters–Tech Access. End repair and A-tailing were performed according to the manufacturer's recommendations. Adapters were ligated using 5 µL of 15 µM stock for each reaction followed by 0.9X AMPure purification. Libraries were amplified with Illumina P5 and P7 primers using NEBNext Ultra II Q5 Master Mix using 6 cycles of amplification. Libraries were purified using 1X AMPure clean up and quantified by Qubit. Following library construction, 500ng of each library was captured with the xGen Pan-Cancer Panel v1.5 following the manufacturer's instructions using xGen Universal Blockers–TS Mix. Hydrophilic Streptavidin Magnetic Beads and NEBNext Ultra II Q5 Master Mix were substituted for the capture and post-capture amplification using 16 cycles of PCR. Libraries were purified, quantified, and pooled for sequencing on an Illumina NovaSeq S4 flow cell.

IDT bioinformatics pipeline

IDT libraries were prepared with xGen Dual Index UMI Adapters—Tech Access which contain 9 bp degenerate unique molecular identifiers (UMIs) downstream of the i7 sample index. To demultiplex Illumina sequencing data containing UMIs, Illumina basecall (BCL) files were used to generate demultiplexed BAM files using Picard v2.9.0[4] IlluminaBasecallsToSam. Prior to running Picard IlluminaBasecallsToSam, Picard v2.9.0 ExtractIlluminaBarcodes was used to determine the barcode for each read using the read structure 151T8B9S8B151T (T = template, B = sample barcode, M = molecular barcode, and S = skip). The UMI bases were not used for downstream analysis since there was not enough raw sequencing depth for consensus analysis. After demultiplexing, FASTQ files were generated using Picard v2.9.0 SamToFastq. FASTQ files were downsampled to an equivalent read count per sample using seqtk[10] v1.0, mapped to hg19 using bwa-mem[3] v0.7.15, and duplicates reads were marked with Picard v2.9.0 MarkDuplicates. Duplicate marked BAM files were used to evaluate target enrichment using Picard v2.9.0 CollectHsMetrics. Variant calling was performed using AstraZeneca VarDict v1.4.8[11], using an allele frequency threshold of ≥0.02, and a minimum of 2 alt reads.

IGT experiment protocol

The iGeneTech AIOnco-seq gene panel was designed to target exon of 113 cancer related genes. The total size of targeted regions was 944,153 bp. 100 ng of human genomic DNA was

sheared on a Covaris S220 focused-ultrasonicator for 180 s (175 W peak incident power, 10% duty factor, and 200 cycles per burst). The resulting fragment-size distribution was about 150~250 bp. End repair, nontemplated dA-tailing, and adapter ligation were performed by using KAPA Hyper Prep Kits. The adapter was generated by annealing of oligonucleotide ACACTCTTTCCCTACACGACGCTCTTCCGATC*T (the * represent a phosphorothioate bond) and oligonucleotide Pho-GATCGGAAGAGCACACGTCTGAACTCCAGTCAC. The ligation product was cleaned up and size-selected by using Beckman Ampure XP Beads (Beckman). The purified ligated product was amplified by using PCR for 7 cycles to generate about 1 µg library.

A mix containing 750 ng whole genome library, 2.5 µg human Cot-1 DNA (Invitrogen), 2.5 µg salmon sperm DNA, and 2000 pmol adapter blocking oligonucleotides, was concentrated to 9 µl, and then heated at 95 ℃ for 5min, and held at 65 ℃ for 5min. A 13 µl of 65 ℃ prewarmed hybridization buffer (iGeneTech) was added to the library. A 7µl of freshly prepared, 65 ℃ prewarmed mixture containing 200 ng biotinylated RNA probes and 20 U SUPERase-In (Invitrogen) was added to the library and mixed by a pipette. After 16 h at 65 ℃, the hybridization mix was added to 50 ul Dynabeads MyOne Streptavidin T1 (Invitrogen), which had been washed three times and resuspended in 200 µl binding buffer (iGeneTech). The binding reaction was rotated on a rotational mixer (10 rpm/min) at 25 ℃ for 30 min, washed once at 25 ℃ for 15 min with 200 µl Wash Buffer I (iGeneTech), and wash three times at 65 ℃ for 15 min with Wash Buffer II (iGeneTech). The beads were resuspended in 20 µl TE Buffer. The post-hybridization PCR was performed to enrich and amplify the target region library. The libraries were sequenced on an Illumina Hiseq 2500 sequencer for 125 bp paired-end sequencing reads.

IGT bioinformatics pipeline

Raw reads were firstly quality trimmed with Trimmomatic[7], using a 8-base-pair sliding-window algorithm with a quality score cutoff of 20, clipping off ends with at least one occurrence of a quality score below 20, and discarding reads that dropped below a length of 40 base-pairs. Adaptors (a1: GATCGGAAGAGCACACGTCT, a2: AGATCGGAAGAGCGTCGTGTAGGGAAAGAGTGT) were removed at the same time. Clean reads were then aligned to the human reference genome (hg19) using BWA MEM algorithm[3] with a seedlength of 20. Optical PCR duplicates were removed using Samtools[12] and locally realigned around indels (java -jar GenomeAnalysisTK.jar -T IndelRealigner) over suspicious intervals determined by RealignerTargetCreator (java -jar GenomeAnalysisTK.jar -T RealignerTargetCreator). The base recalibration table was generated to compensate for systematic errors in basecalling confidences using BaseRecalibrator (java -jar GenomeAnalysisTK.jar -T BaseRecalibrator). VarScan[13] is a platform-independent mutation caller for targeted, exome, and whole-genome resequencing data generated on Illumina, SOLiD, Life/PGM, Roche/454, and similar instruments. The newest version is VarScan2. We use

VarScan2 mpileup2cns algorithm to call single nucleotide polymorphisms (SNPs), small insertions and deletions (InDels) from short-read sequencing data aligned against a reference genome (java -jar VarScan2.jar mpileup2cns --min-var-freq 0.01 --p-value 0.05). The detected variants were finally saved as VCF files.

ILM experiment protocol

DNA were processed according to the TruSight Tumor 170 Reference Guide[14], briefly samples were sheared using Covaris to approximately 90-250bp. Following shearing DNA fragments were end-repaired and A-tailed in a single reaction, followed by ligation to an universal adapter. Post-ligation clean-up was performed using SPRI beads and then libraries were indexed using unique dual indexes by PCR. Target regions were captured using an overnight hybridization to biotinylated target specific oligos which cover 533Kb of genomic targets across 154 genes, followed by capture with streptavidin magnetic beads. A second hybridization and capture reaction were performed followed by PCR amplification using the universal primers compatible with Illumina's sequencing flowcells. Libraries were normalized using bead-based normalization before being pooled in equal parts and sequenced, 10 samples per flowcell, on NextSeq v2 high-output flowcell. Sequencing reads were 2 x 101bp with 8bp dual indexed reads. Five independent labs were recruited as the test labs for ILM panel. One test lab was excluded from performance analysis due to an extensive delay in data generation and some deviation from the experiment protocol.

ILM bioinformatics pipeline

For Pan Cancer sample testing analysis was performed using the standard TruSight Tumor 170 pipeline available on the BaseSpace Sequence Hub. Briefly, high level sequencing run metrics are evaluated to generate a Run QC Metrics report. Next reads are converted into the fastq format using bcl2fastq[1], adapters are trimmed and then reads are aligned to the human genome version hg19 using the iSAAC aligner[15]. Indel realignment was performed and then candidate variants were identified using the Pisces variant caller[16], with a fixed lower limit cutoff for variant allele fraction of at least 2.6%. Variant calls are further compared against a baseline of normal samples to remove systematic false positives.

QGN experiment protocol

The Human Comprehensive Cancer QIASeq DNA Panel is a targeted enrichment panel that enriches genes using single primer extension and constructs libraries using integrated unique molecular indices (UMIs). The panel uses 11,311 primers in a single tube mix to enrich over 0.83 Mb region across full coding areas of 275 cancer genes. The selected genes harbor mutations that are commonly involved in cancer development and progression. Genomic DNA samples were first fragmented, end repaired and A-tailed within a single, controlled multi-

enzyme reaction. The prepared DNA fragments were then ligated at their 5' ends with an Illumina sequencing platform-specific adapter containing a 12-bases fully random sequence of UMIs and sample index. Therefore, each original DNA molecule in the sample received a unique UMI sequence during ligation prior to target enrichment and library amplification PCR. This allowed the correction of errors associated with PCR amplification and sequencing.

Target enrichment with Human Comprehensive Cancer QIASeq DNA Panel was performed post-UMI assignment to ensure that targeting regions of DNA molecules containing UMIs were sufficiently and uniformly enriched in the sequenced library. For enrichment, ligated DNA molecules were subject to six cycles of targeted PCR using one region-specific primer and one universal primer complementary to the adapter. After enrichment PCR, nineteen cycles of Universal PCR were carried out to further amplify the target enriched library and added platform specific adapter sequences and additional sample indices. Finished libraries were checked on Bioanalyzer for proper size distribution and library concentration was measured by QIAseq Library Quantification system. Libraries were normalized to 5 nM and pooled together according to quantified library concentration. Pooled libraries were sent out to Illumina for sequencing on NovaSeq with a custom read 1 sequencing primer. Libraries within a test site were loaded on each lane during sequencing and sequenced FASTQ files were downloaded from BaseSpace for further analysis.

QGN bioinformatics pipeline

Variants in the QIAseq Targeted DNA panel were detected by QIAGEN's UMI-aware variant caller smCounter2[17]. The variant calling pipeline begins with read processing steps that trim the exogenous sequences such as PCR and sequencing adapters and UMI. Short trimmed reads (< 40 bases) are discarded. The trimmed reads are mapped to the reference genome with BWA-MEM[3], followed by filtering of poorly mapped reads and soft-clipping of gene-specific primer sequence. A UMI with small read count is combined with a larger UMI family if the two UMIs are within one edit distance and the corresponding 5' positions of aligned R2 reads (i.e. at the random fragmentation site) are within 3 bp. After UMI clustering, the aligned reads (BAM format) are sent forward to variant calling.

At the core of smCounter2 is a statistical test to determine whether the allele frequency of the putative variant is significantly above the background error rate, which was carefully modeled using an independent dataset. smCounter2 treats variant calling as a hypothesis testing problem. The null hypothesis (H_0) is that all non-reference UMIs are from background errors and the alternative hypothesis (H_a) is that non-reference UMIs are from the real variant. Suppose there are n UMIs covering a site and k of them have the same non-reference allele. Under H_0, k follows a Binomial distribution $Bin(n, p)$, where p is the background error rate.

We fit a Beta distribution with parameters a and b on p. The marginal distribution of k given (n, a, b) is Beta-binomial under H_0 and the P-value can be computed by numerical integration. smCounter2 reports $Q = \min(200, -\log_{10} P)$ as the variant quality score, i.e. QUAL in the output VCF file. Higher quality score indicates stronger UMI support for a variant. By default, smCounter2 requires $Q \geq 6.0$ for all types of variants. If 0.5-1% indels need to be detected with high sensitivity, we recommend lowering the quality threshold to 2.5 to account for overestimation of indel error rates.

A key step in the above procedure is the estimation of the background error rate distribution. This was achieved by sequencing an NA12878 sample with very high input amount and sequencing depth. After excluding known variants, we calculated the error rates by base substitution at each site and then fit a Beta distribution for each base substitution type if enough errors are observed.

The statistical model allows smCounter2 to set a constant quality score threshold at 6.0. This threshold controls false positive rate at a low level across a range of UMI depths, as demonstrated by a series of *in silico* down-sampling experiments and in multiple sequencing runs with various UMI depths and sequencing depths. The detection limit of smCounter2 and QIAseq Targeted DNA Panels is about 0.5% in allele frequency.

ROC experiment protocol

The panel design utilized in the study is 170907_HG38_SEQC2_PHC_EZ. This is a research panel, not intended for commercial development. The panel consists of coding sequence (CDS) from 33 cancer genes from SEQC Pan Cancer Gene list and CDS regions overlapping the Accugenomics controls. Additional targets were derived from the Roche Avenio ctDNA Panels. The panel consists of 45 genes in total (~131 kb of targets). The panel also targets 730 putative structural variant breakpoints derived from the 10X cell line sequencing (~206 kb of targets).

The ROC panel utilized an enzymatic fragmentation library prep followed by a hybridization-based workflow. The extracted DNA sample (100 ng) was fragmented using the KAPA HyperPlus Library Preparation Kit, and then ligated to Ilumina-compatible, unique-dual-index (UDI) sequencing library adapters (IDT). After the ligation, the DNA library was amplified using the KAPA HiFi HotStart Ready Mix. Amplified libraries were quantified with a fluorometric method, and the maximum available amount of library (>= 0.5 ug and <= 2.5 ug) was utilized for capture. The sample was incubated overnight at 47 C with the gene panel (170907_HG38_SEQC2_PHC_EZ), which consisted of biotinylated DNA probes designed to capture the genes and regions of interest. Universal Blocking Oligos were utilized in the hybridization to prevent cross-hybridization between sequencing adapters and to increase

capture specificity. The desired DNA-probe complexes were then captured on streptavidin beads, and after a series of washes, first at 47 C and then at room temperature, the samples were amplified using ligation mediated PCR (LM-PCR). The final product of the workflow was enriched libraries ready for sequencing. The final sequencing libraries were sequenced (2x150 bp) using the Illumina NovaSeq sequencing platform.

Four independent labs were recruited to test the ROC panel. Initial sequencing data QC analysis led to the exclusion of one test lab from performance analysis for ROC. Shorter fragments, which resulted from over fragmentation during library preparation, indicated a likely deviation from the experiment protocol.

ROC bioinformatics pipeline

Two NovaSeq runs were provided by Illumina via BaseSpace (RUN1=SeqC2_ROC1_ST_16_17_18_19; RUN2= SeqC2_ROC1_ST16_17_18_19). BCL files were converted to unaligned BAM files using instructions provided by IDT (https://www.idtdna.com/pages/products/next-generation-sequencing/adapters/xgen-dual-index-umi-adapters-tech-access). Following the IDT tech note, Picard 2.18.3[4] ExtractIlluminaBarcodes and IlluminaBasecallsToSam were used to create the unaligned BAM files. The UMI for each fragment is stored in the RX tag in the BAM file. The unaligned BAM files produced by this workflow have been uploaded to the Stanford SFTP website.

GATK 4.0.6.0[6] SortSam was used to sort the unaligned BAM by queryname. GAKT 4.0.6.0 MarkIlluminaAdapters was used to find and tag adapter sequences. Picard 2.18.4 SamToFastq (CLIPPING_ATTRIBUTE=XT CLIPPING_ACTION=2) was used to create FASTQ files from the BAM files with marked adapters. FASTP 0.19.3[18,19] was used to do quality trimming, additional adapter trimming, polyG trimming, read correction (-g -W 5 -q 20 -u 40 -x -3 -g --poly_g_min_len 3 -l 75 –c). Quality and adapter trimmed reads were mapped with BWA 0.7.17 to the hg38 genome, and merged with the unaligned BAM using Picard 2.18[4] MergeBamAlignment (PAIRED_RUN=true SORT_ORDER="queryname" ALIGNED_READS_ONLY=true CLIP_ADAPTERS=true ADD_MATE_CIGAR=true MAX_INSERTIONS_OR_DELETIONS=-1 PRIMARY_ALIGNMENT_STRATEGY=MostDistant UNMAPPED_READ_STRATEGY=COPY_TO_TAG ALIGNER_PROPER_PAIR_FLAGS=true UNMAP_CONTAMINANT_READS=true ADD_PG_TAG_TO_READS=false). UMI families were identified using fgbio[20] 0.6.0 GroupReadsByUmi (--strategy=adjacency –edits=1) and then consensus reads were generated using fgbio 0.6.0 Call MolecularConsensus Reads, requiring at least 2 read pairs in each UMI family (--error-rate-post-umi 40 --error-rate-pre-umi 45 --output-per-base-tags false --min-reads 2 --max-reads 50 --min-input-base-quality 20). Final FASTQ files

were generated by using GATK 4.0.6.0 SamToFastq to convert the consensus BAM files to paired FASTQ files.

BWA 0.7.17[3] was used to map the N=2 consensus reads to the hg38 genome. When subsampling was performed seqtk[10] was utilized to perform the subsampling on the input FASTQ files, using the same random seed for read 1 and read2. After read alignment, GATK 4.1.0.0 was used to convert to BAM format (SamFormatConverter), fix mate pair information (FixMateInformation) and then sorted by position (SortSam). Base quality score recalibration (BQSR) was performed using GATK 4.1.0.0 BaseRecalibrator and ApplyBQSR. Common variant sites used by BaseRecalibrator included those supplied in the GATK hg38 resource bundle (dbsnp_146.hg38.vcf.gz,1000G_omni2.5.hg38.vcf.gz,1000G_phase1.snps.high_confidence.hg38.vcf.gz,Mills_and_1000G_gold_standard.indels.hg38.vcf.gz) and the gnomAD hg38 variant sites file (uploaded to Stanford SFTP site: /Users/fda_user/SEQC2tmp/SEQC2_disk2/Roche_hg38_WES1_WES2_WES3/analysis_pipeline/gnomad.genomes.r2.0.1.sites.GRCh38.AF_only.fixed.vcf.gz)

After base quality score recalibration, hard and soft-clipped read pairs were removed from the recalibrated BAM. An awk command and command-line utilities were used to identify clipped reads (awk -F '\t' '($6 ~ /H|S/)' | cut -f 1 | sort -V –u) and product a list of read IDs. Those reads were removed using GATK 4.1.0.0 FilterSamReads. GATK 4.1.0.0 Mutect2[5] was then used to call variants in the CDS regions of the capture panel:

```
java -Xms16g -Xmx16g -jar gatk-package-4.1.0.0-local.jar Mutect2
    --input input.bam
    --output output.vcf
    --reference hg38.fa
    --tumor sample_id
    --intervals 170907_HG38_SEQC2_PHC_EZ_primary_targets.CCDS_only.bed
    --intervals 170907_HG38_SEQC2_PHC_EZ_capture_targets.CCDS_only.bed
    --min-base-quality-score 20
    --minimum-mapping-quality 30
    --native-pair-hmm-threads 12
    --annotation-group StandardMutectAnnotation
    --create-output-variant-index true
    --genotype-germline-sites true
    --annotate-with-num-discovered-alleles true
    --pcr-indel-model AGGRESSIVE
    --max-reads-per-alignment-start 0
```

TFS experiment protocol

The Oncomine Comprehensive Assay DNA v3C – Chef Ready Kit[21] was used to generate libraries for next-generation sequencing on the Ion Torrent S5 platform. This assay enables analysis of variants across 146 genes and covers 350,350 bp of DNA target sequence. It typically is used in combination with an accompanying RNA assay bringing the gene total to 161, but the study used only the DNA assay with the RNA component omitted. The gene content of the Oncomine Comprehensive Assay was prioritized to detect relevant somatic variants in solid tumors with associations to published evidence including the indication statements of approved cancer drugs, consensus clinical treatment guidelines, and the enrollment criteria of oncology clinical trials. Purified DNA was extracted using the RecoverAll Multi-Sample RNA/DNA Isolation Workflow[22]. Four DNA samples were used to prepare duplicate libraries on each of two runs using the Ion Chef Instrument. An input of 10ng per primer pool of DNA was used, and libraries were generated following the manufacturer's instructions in the Oncomine Comprehensive AssayUser Guide[23] and the Ion AmpliSeq Library Preparation on the Ion Chef User Guide[24]. A total of 8 DNA sample libraries were combined for template preparation on the Ion Chef Instrument using the Ion 540 Kit-Chef[25]. Sequencing was performed with the Ion S5 XL System[26] and the Ion 540 Chip[27].

TFS bioinformatics pipeline

Signal processing and base calling were performed using Torrent Suite Software 5.8[28] using default parameters for the Oncomine Comprehensive Assay. The signal processing step consists of modeling the pH dynamics on the semiconductor surface taking account of the varying local pH in each individual sensor coming from the different reagent flows across the chip and from any nucleotide incorporation that may be happening over each sensor[29]. The base calling step consists of taking the estimated levels of nucleotide incorporation for each read and each nucleotide flow, and modeling the de-phasing process whereby some templates within each clonally-amplified population run ahead or behind in terms of their nucleotide incorporation. During the base calling process, sample-specific barcodes and 3' adapters are annotated.

After completion of primary analysis with Torrent Suite Software 5.8[28], reads were uploaded to Ion Reporter Software 5.6[30] for subsequent processing. Reads were aligned with the TMAP module[31], which uses the BWA fastmap routine to map reads and applies post-processing of the alignments to optimize for technology-specific error patterns. After alignment, variant calling was performed with Torrent Variant Caller (TVC)[32], a variant calling module optimized for Ion Torrent data. The TVC module takes as input the aligned reads and uses a modified version of freebayes to generate a very permissive list of candidate de-novo alleles to be evaluated. The list of de-novo alleles is merged with a list of pre-defined hotspot

alleles representing variants known to be highly recurrent in cancers. At this stage the list of candidates to evaluate is filtered against a list of pre-defined recurrent systematic errors, to suppress false positives. The remaining alleles are evaluated in a statistical likelihood model that compares the observed flow signals for all of the aligned reads with the flow signals that would be expected under reference and non-reference hypotheses. The use of flow signals leads to significant improvements in variant calling compared to variant calling approaches that rely on base calls alone. At each position evaluated, the posterior likelihood of each evaluated allele's frequency is assessed to determine if the null hypothesis that the allele frequency is less than or equal to a particular threshold can be rejected. For the Oncomine Comprehensive Assay the default thresholds used for SNVs and indels is 2.5 percent. No variants are called below the threshold and variants can be called just above the threshold if the statistical evidence is sufficiently strong. Finally, a series of post-calling filters are applied to variant calls to filter out situations where the statistical model of flow signals is not a good fit for the observed data, and to eliminate potential artifacts where a variant appears on only one strand in regions with coverage on both strands, or in only one amplicon in regions where more than one amplicon spans the variant.

The Ion Torrent sequencing chip is comprised of millions of wells each containing a bead. Each bead contains millions of copies of the target (that is, template) molecule (i.e. cDNA), and represents one sequencing read. As a result of cDNA amplification prior to sequencing, each target base is typically represented by multiple beads. During the sequencing of targeted Ampliseq panels, with the Oncomine Comprehensive Assay for this manuscript, nucleotide flows can synchronize across reads and interact with the sequencing error. This can result in consistent mismatch in reads from the underlying template (or target) DNA molecule. These errors are systematic, and are called as Strand Specific Error (SSE) as they are generally present on only one strand. For the variant profiling of the low allele frequency target variants, these systematic errors can result in false-positive variant calls, and should be identified to suppress. The SSE error profile for the Oncomine Comprehensive Assay was derived by running wild-type DNA samples that are known to be free of somatic variants. Collection of such SSE profiles is known as blacklist and is provided as one of the input files that is used in variant calling. The TVC module deploys a complex series of filters to remove SSE variants to minimize false positive calls among somatic variants.

Exclusion of spike-in variants for IDT

AstraZeneca VarDict[11] v1.4.8 was used for variant calling in IDT's bioinformatics pipeline. The specific version of Vardict has a default filter called NM4.25 to discard reads with mean number of mismatches greater than or equal to 4.25. This means that if multiple variants are co-located with each other, these regions may have reads being thrown out due to this filter resulting in

missed variant calls. The list of spike-in variants was filtered for variants that were co-located with each other. If at least four variants were found to be co-located within 151bp, then these mutations were flagged and removed from the list. The filtered list of spike-in variants was adopted to assess the panel IDT's sensitivity.

Exclusion of spike-in variants for QGN

Some primers in the QIAGEN panel overlap with one or more AcroMetrix Spike-in variants. These primers may bind to the non-reference genome with lower efficiency than they normally would to the reference genome. As a result, nearby downstream variants may have insufficient read evidence and therefore missed by the variant caller. Although this problem can occur in any sample, usually at a low rate, it is greatly magnified by the artificially dense design of AcroMetrix Spike-in variants. For this reason, QIAGEN, agreed by SEQC2, decided to exclude the affected variants from data analysis for the QIAGEN panel. The details of QIAGEN's variant exclusion procedure are elaborated below.

First, for each AcroMetrix variant in the panel, all supporting primers were searched. Because fragment lengths are typically less than 300bp in QIAseq panels, primers whose 3' end is within 300bp upstream from the end of the variant were selected. These primers should generate most or all of the reads that cover the target variant. Because nearby AcroMetrix variants are located on the same DNA fragment (haplotype) by design, primers overlapping with other AcroMetrix variant(s) may not effectively capture the target AcroMetrix variants. These supporting primers were thus considered defected if they overlap with other AcroMetrix variants.

If one or more supporting primers were defected, the target AcroMetrix variant was excluded from downstream data analysis. Although other supporting primers should work normally, the failure of one or more primers may trigger the "Primer Bias" filter in smCounter2, which rejects variants whose read evidence is unevenly distributed among supporting primers.

Exclusion of spike-in variants for TFS

Analytical sensitivity assessment of TFS required pre-filtering to exclude variants from un-callable positions due to the panel design. Variants from these positions were not considered for assessment and excluded from the list of known (or expected) spike-in variants. Firstly, any variant that was not present within the target genomic regions of TFS was excluded. Panel design also includes known systematic error positions, (also known as 'blacklist' positions described above in Subsection "TFS bioinformatics pipeline"), which are masked during the variant calling. Spike-in variants that overlap with blacklist positions were also excluded. Due to the high density of spike-in variants, some variants may overlap with amplicon primer regions.

These amplicons generate read coverage, i.e. are included in the sequencing reads, however only reference calls will be present since no test (spike-in) DNA would be amplified. We identified and excluded spike-in variants from the sensitivity assessment that fall within the amplicons of such primers.

Component analysis of TMB mean squared deviation (MSD)

For a group of TCGA samples with TMB rates close to each other and thus close to their mean value, the average TMB rate can be used to approximate the individual ones within the group. This leads to a modified MSD, denoted as MSD'. For a given panel, the MSD' is calculated as

$$MSD' = \frac{\sum_{i=1}^{n}(Panel.TMB_i - avg(TCGA.TMB))^2}{N}$$

Still, *i* is an individual tumor sample and N is the number of the samples in the group.
To simplify the analysis of MSD, let $x_i = TCGA.TMB_i$, $y_i = Panel.TMB_i$, and $\mu_x = avg(TCGA.TMB)$, then

$$MSD = \frac{\sum_{i=1}^{n}(y_i - x_i)^2}{N} \text{ and } MSD' = \frac{\sum_{i=1}^{n}(y_i - \mu_x)^2}{N}$$

Furthermore,

$$\sum_{i=1}^{n}(y_i - \mu_x)^2 = \sum_{i=1}^{n}(y_i - x_i + x_i - \mu_x)^2$$

$$= \sum_{i=1}^{n}(y_i - x_i)^2 + \sum_{i=1}^{n}(x_i - \mu_x)^2 + 2\sum_{i=1}^{n}(y_i - x_i)(x_i - \mu_x)$$

As we know, $(x_i - \mu_x)$ is a random number sequence and $\sum_{i=1}^{n}(x_i - \mu_x) = 0$. We can assume that $\sum_{i=1}^{n}(y_i - x_i)(x_i - \mu_x) = 0$ as $(x_i - \mu_x)$ is independent from $(y_i - x_i)$ that is also a random number sequence. This assumption can be verified and viewed as a result of the random and independent distribution of mutations within each sample.

Thus,

$$MSD' = \frac{\sum_{i=1}^{n}(y_i - x_i)^2}{N} + \frac{\sum_{i=1}^{n}(x_i - \mu_x)^2}{N}$$

$$MSD' = MSD + \frac{\sum_{i=1}^{n}(x_i - \mu_x)^2}{N} = MSD + {\sigma_x}^2$$

On the other hand,

$$MSD' = (\mu_y - \mu_x)^2 + \frac{\sum_{i=1}^{n}(y_i - \mu_y)^2}{N} = (\mu_y - \mu_x)^2 + {\sigma_y}^2$$

Finally,

$$MSD' = MSD + {\sigma_x}^2 = (\mu_y - \mu_x)^2 + {\sigma_y}^2$$

$$MSD = (\mu_y - \mu_x)^2 + ({\sigma_y}^2 - {\sigma_x}^2)$$

To conclude, MSD is consisted of two components: squared mean bias and difference in variance.

Reference:

1. Illumina. bcl2fastq2 Conversion Software v2.20. https://support.illumina.com/downloads/bcl2fastq-conversion-software-v2-20.html. Accessed 30 Jan. 2020.

2. The Agilent Genomics NextGen Toolkit. https://www.agilent.com/en/product/next-generation-sequencing/hybridization-based-next-generation-sequencing-ngs/ngs-software/agent-232879.

3. Li H. Aligning sequence reads, clone sequences and assembly contigs with BWA-MEM. ArXiv13033997 Q-Bio. 2013; http://arxiv.org/abs/1303.3997

4. Broad Institute. Picard Tools. http://broadinstitute.github.io/picard/. Accessed 22 Dec. 2017.

5. Broad Institute. MuTect2. https://software.broadinstitute.org/gatk/documentation/tooldocs/3.8-0/org_broadinstitute_gatk_tools_walkers_cancer_m2_MuTect2.php

6. McKenna A, Hanna M, Banks E, Sivachenko A, Cibulskis K, Kernytsky A, et al. The Genome Analysis Toolkit: A MapReduce framework for analyzing next-generation DNA sequencing data. Genome Res. 2010;20(9):1297–303.

7. Bolger AM, Lohse M, Usadel B. Trimmomatic: a flexible trimmer for Illumina sequence data. Bioinformatics. 2014;30(15):2114–20.

8. Wang K, Li M, Hakonarson H. ANNOVAR: functional annotation of genetic variants from high-throughput sequencing data. Nucleic Acids Res. 2010;38(16):e164–e164.

9. Cingolani P, Platts A, Wang LL, Coon M, Nguyen T, Wang L, et al. A program for annotating and predicting the effects of single nucleotide polymorphisms, SnpEff. Fly (Austin). 2012;6(2):80–92.

10. Li H. lh3/seqtk. 2020; https://github.com/lh3/seqtk

11. Lai Z, Markovets A, Ahdesmaki M, Chapman B, Hofmann O, McEwen R, et al. VarDict: a novel and versatile variant caller for next-generation sequencing in cancer research. Nucleic Acids Res. 2016;44(11):e108.

12. Li H, Handsaker B, Wysoker A, Fennell T, Ruan J, Homer N, et al. The Sequence Alignment/Map format and SAMtools. Bioinformatics. 2009;25(16):2078–9.

13. Koboldt DC, Zhang Q, Larson DE, Shen D, McLellan MD, Lin L, et al. VarScan 2: Somatic mutation and copy number alteration discovery in cancer by exome sequencing. Genome Res. 2012;22(3):568–76.

14. Illumina. TruSight Tumor 170 Reference Guide. https://support.illumina.com/content/dam/illumina-support/documents/documentation/chemistry_documentation/trusight/tumor-170/trusight-tumor-170-reference-guide-1000000024091-02.pdf. Accessed 31 Jan. 2020.

15. Raczy C, Petrovski R, Saunders CT, Chorny I, Kruglyak S, Margulies EH, et al. Isaac: ultra-fast whole-genome secondary analysis on Illumina sequencing platforms. Bioinformatics. 2013;29(16):2041–3.

16. Illumina. Pisces. Illumina; 2019; https://github.com/Illumina/Pisces

17. Xu C, Gu X, Padmanabhan R, Wu Z, Peng Q, DiCarlo J, et al. smCounter2: an accurate low-frequency variant caller for targeted sequencing data with unique molecular identifiers. Bioinformatics. 2019;35(8):1299–309.

18. Chen S, Zhou Y, Chen Y, Gu J. fastp: an ultra-fast all-in-one FASTQ preprocessor. Bioinformatics. 2018;34(17):i884–90.

19. OpenGene. fastp. OpenGene - Open Source Genetics Toolbox; 2020; https://github.com/OpenGene/fastp

20. fulcrumgenomics, fulcrumgenomics, fulcrumgenomics. fgbio. Fulcrum Genomics; 2020; https://github.com/fulcrumgenomics/fgbio

21. Thermo Fisher Scientific, "Oncomine™ Comprehensive Assay v3C - A35806. http://www.thermofisher.com/order/catalog/product/A35806. Accessed 30 Jan. 2020.

22. RecoverAll™ Multi-Sample RNA/DNA Isolation Workflow A26069. http://www.thermofisher.com/order/catalog/product/A26069. Accessed 30 Jan. 2020.

23. Oncomine™ Comprehensive Assay v3 User Guide - MAN0015885. https://assets.thermofisher.com/TFS-Assets/LSG/manuals/MAN0015885_OncomineComprehensiveAssay_v3_UG.pdf. Accessed 30 Jan. 2020.

24. Thermo Fisher Scientific. Ion AmpliSeq™ Library Preparation on the Ion Chef™ System User Guide MAN0013432. https://assets.thermofisher.com/TFS-Assets/LSG/manuals/MAN0013432_Ion_AmpliSeq_Library_Prep_on_Ion_Chef_UG.pdf. Accessed 30 Jan. 2020.

25. Thermo Fisher Scientific. Ion 540™ Kit-Chef A30011. http://www.thermofisher.com/order/catalog/product/A30011. Accessed 30 Jan. 2020.

26. Thermo Fisher Scientific. Ion S5^{TM} XL System A27214. http://www.thermofisher.com/order/catalog/product/A27214. Accessed 30 Jan. 2020.

27. Thermo Fisher Scientific. Ion 540^{TM} Chip Kit A27766. http://www.thermofisher.com/order/catalog/product/A27765. Accessed 30 Jan. 2020.

28. Thermo Fisher Scientific. Torrent Suite Software. Ion Torrent; 2019; https://github.com/iontorrent/TS

29. Rothberg JM, Hinz W, Rearick TM, Schultz J, Mileski W, Davey M, et al. An integrated semiconductor device enabling non-optical genome sequencing. Nature. 2011;475(7356):348–52.

30. Thermo Fisher Scientific. Ion Reporter Software. https://www.thermofisher.com/us/en/home/life-science/sequencing/next-generation-sequencing/ion-torrent-next-generation-sequencing-workflow/ion-torrent-next-generation-sequencing-data-analysis-workflow/ion-reporter-software.html. Accessed 16 Oct. 2019.

31. Thermo Fisher Scientific. TMAP - Torrent Mapper. https://github.com/iontorrent/TS. Accessed 16 Oct. 2019.

32. Thermo Fisher Scientific. Torrent Variant Caller. http://updates.iontorrent.com/tvc_standalone/. Accessed 16 Oct. 2019.
