# Supplementary material for: Cross-oncopanel study reveals high sensitivity and accuracy with overall analytical performance depending on genomic regions
Source: Genome Biol. 2021 Apr 16;22:109. doi: 10.1186/s13059-021-02315-0 (PMC8051090; doi:10.1186/s13059-021-02315-0)
Supplement: Supplementary file 3 — Additional file 3: Fig. S1. Concordance of average cross-lab reproducibility and intra-lab reproducibility in Phred scale across VAF ranges for samples A and C. Fig. S2. Comparison of sensitivity of different variant types across VAF ranges. Fig. S3. Estimation of the false positive rate by three methods. Fig. S4. Low reproducibility of false positive calls across library replicates. Fig. S5. Scatter plot of panel TMB mean × panel_size (x-axis) and adjusted panel TMB variance × panel_size2 (y-axis). [file 13059_2021_2315_MOESM3_ESM.pdf]

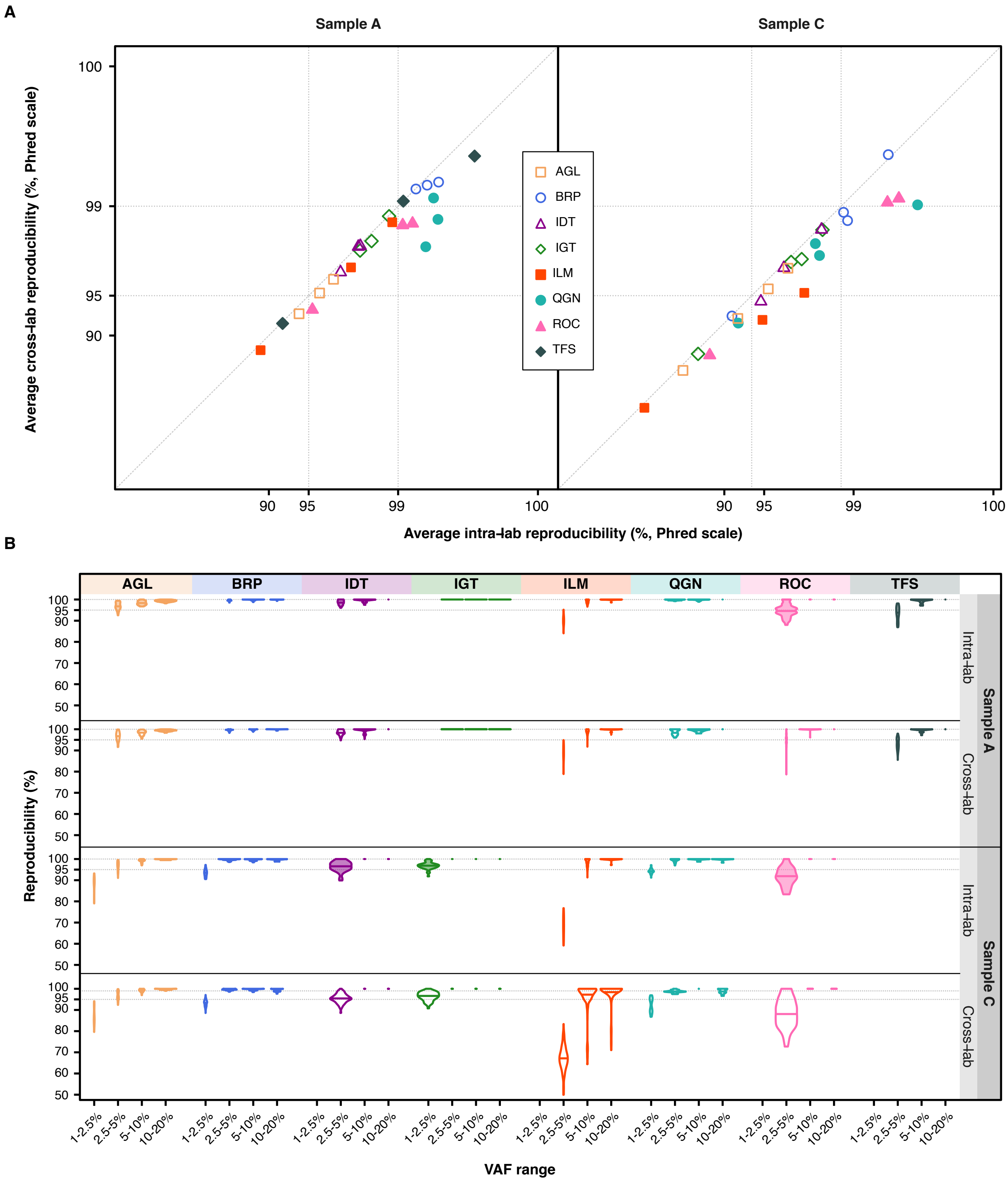

Fig. S1: Concordance of average cross-lab reproducibility and intra-lab reproducibility in PHRED scale across VAF ranges for samples A and C. (A) Concordance of average cross-lab reproducibility and intra-lab reproducibility in PHRED scale across VAF ranges for samples A and C. (B) Violin plots of cross-lab and intra-lab reproducibility in PHRED scale for samples A and C in each VAF range.

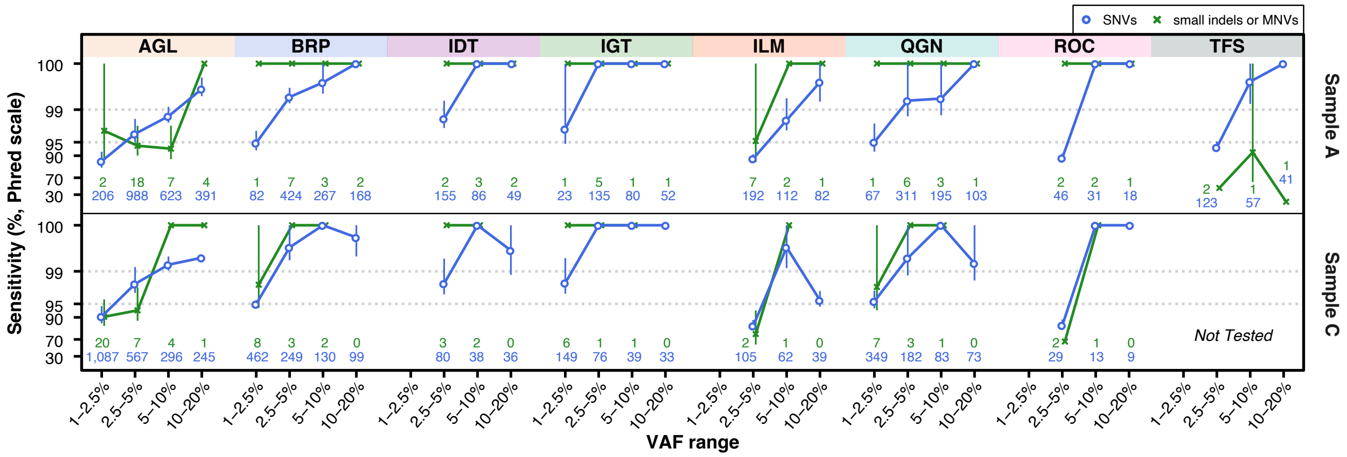

Fig. S2: Comparison of sensitivity of different variant types across VAF ranges. Line plot of sensitivity in Phred scale with error bars across VAF ranges for samples A and C of SNVs (in blue open circle) and others (small indels or MNVs in green "x"). Numbers of variants are listed at the bottom.

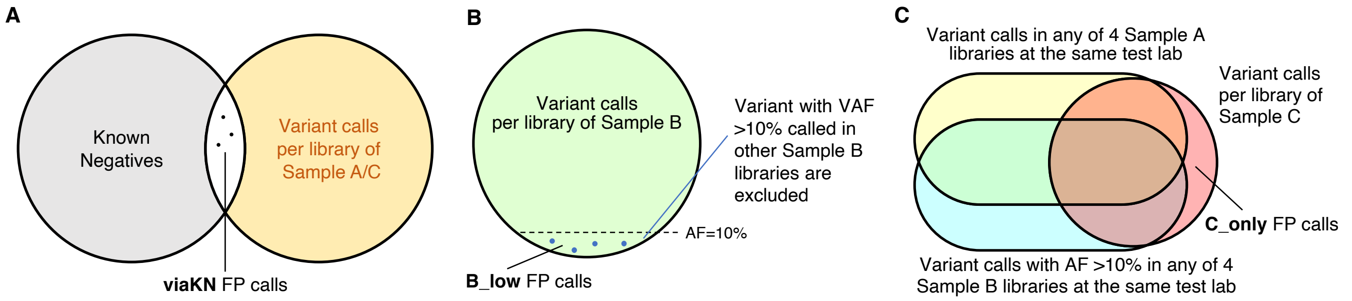

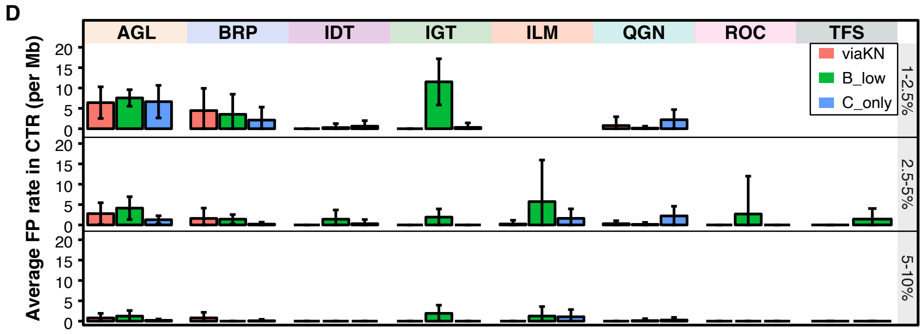

E

	CTR	HC_CR beyond CTR	Rest
AGL	2,017,891	288,027	5,319,245
BRP	822,595	108,588	141,055*
IDT	295,787	48,223	455,580
IGT	260,536	25,447	658,170
ILM	348,802	36,394	141,379
QGN	603,264	74,196	159,210
ROC	93,129	8,942	47,303
TFS	174,190	17,176	97,953

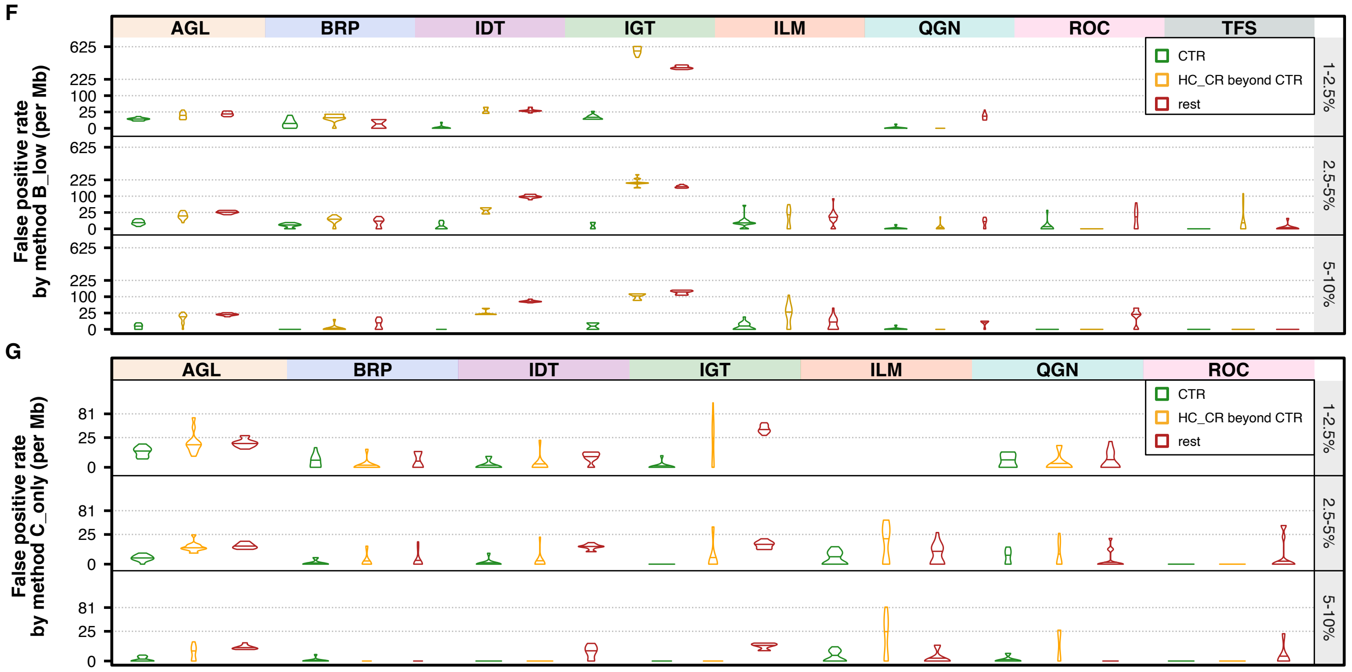

Fig. S3: Estimation of false positive rate by three methods. Sketch illumination of three FP call determination methods: (A) viaKN, (B) B_low, and (C) C_only. (D) Bar charts of FP rate by all three methods in the CTR across VAF ranges for each panel. (E) List of covered bases by each panel in three sub-regions: CTR, HC_CR beyond CTR, and the rest of the panel. *BRP bioinformatics pipeline only reports variants on restricted region (see Additional file 2: Supplementary Methods). The actually calling region size of this "Rest" region of BRP is 141,055. (F) Violin plots of FP rate estimated by method B_low across VAF ranges for each panel in three sub-regions. (G) Violin plots of FP rate estimated by method C_only across VAF ranges for each panel in sub-regions.

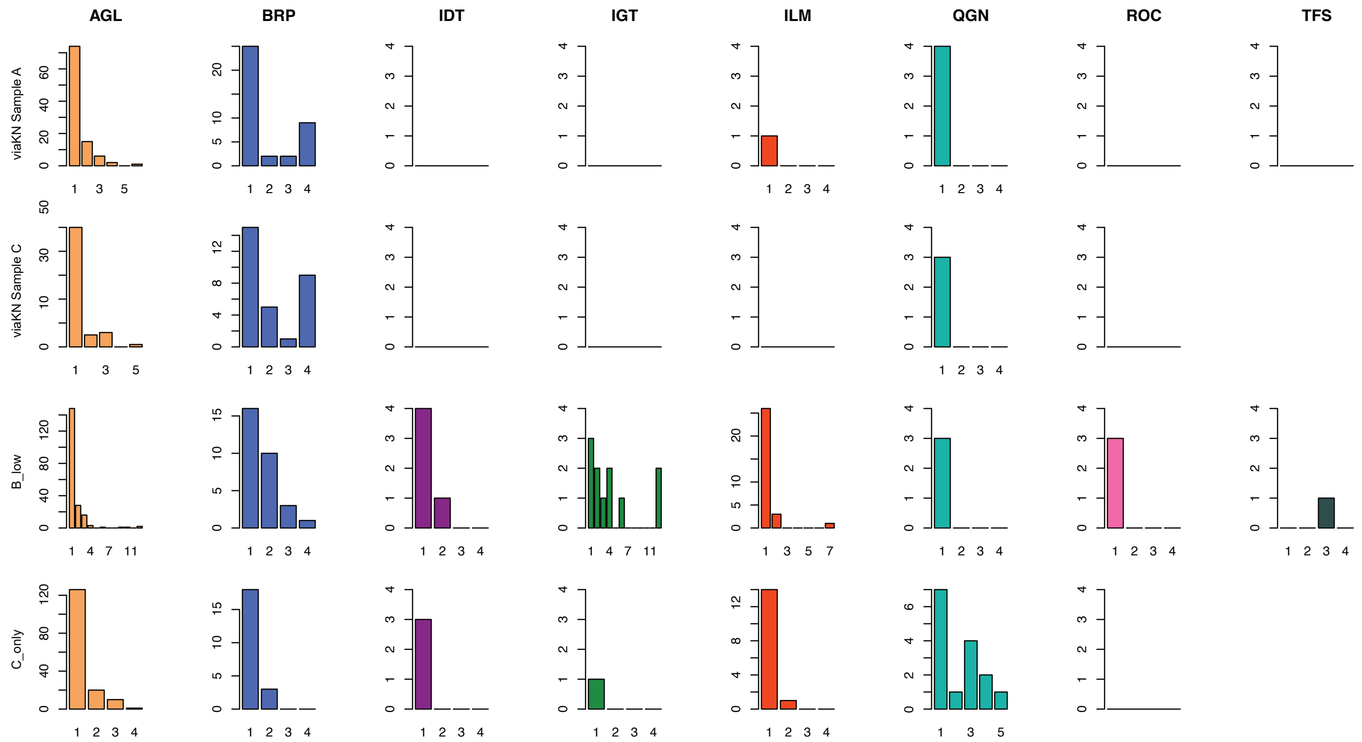

Fig. S4: Low reproducibility of false positive calls across library replicates. The bar charts show the number (y-axis) of false positive variants called by how many library replicates (x-axis) for each panel, sample, and FP method. The false positive variants include both SNVs, small indels, and MNVs that were called within the CTR. The total number of library replicates per sample for each panel was 12 except ILM had 16 library replicates. Except very few of them (5 out of about 200 for AGL and 3 out of 11 for IGT identified by B_low), all false positive calls were detected in less than 50% of library replicates. Most of them were called in only one library replicate. All three false positives repeatedly called by IGT in Sample B were 3 bp deletions. Each was also called in Sample A with a similar number of library replicates and a strikingly similar VAF (the absolute difference was less than 0.1%). They were likely caused by some systematic errors or biases as these two samples were not related. Similarly, three false positives called by AGL in Sample B were called in Sample A, each with a similar number of library replicates. The absolute differences in VAF were 0.2%, 0.8%, and 0.6%. Likely these false SNV calls were caused by some systematic errors or biases. The other two false positives were called in Sample B with a low average VAF, 1.3% and 2.0%, respectively. They were not called in any Sample A or Spike-in libraries. The Spike-in sample used Sample B as the genomic background. It was sequenced to the same targeted depth as Sample B. Among all Sample B and Spike-in libraries, these two false positives were called in less than 50% of library replicates. This adjusted low reproducibility supports the classification of them as false positives.

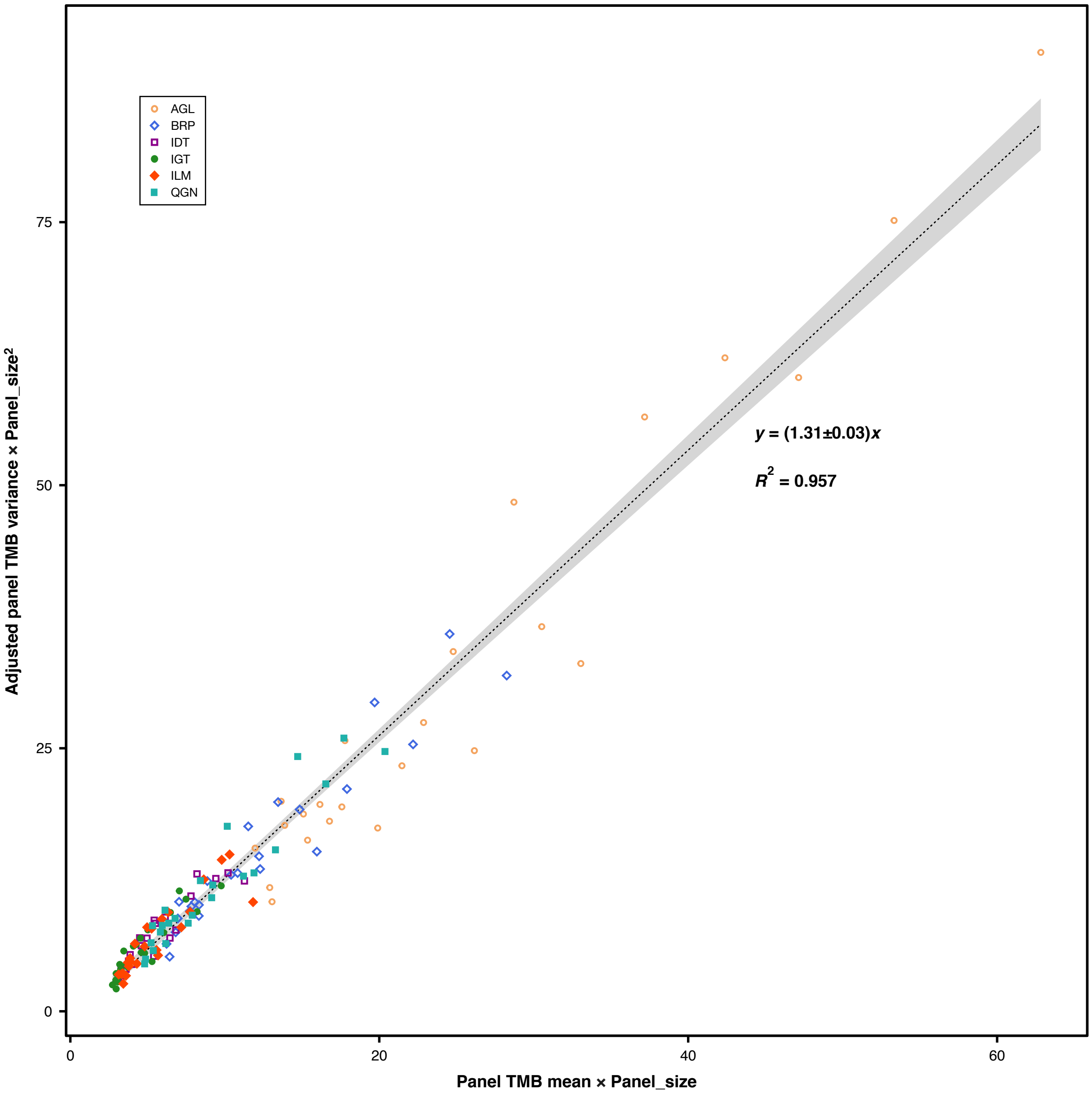

Fig. S5: Scatter plot of panel TMB mean × panel_size (x-axis) and adjusted panel TMB variance × panel_size2 (y-axis). Each dot is calculated from a group of 100 samples with similar TMB estimated in the overlapping region between TCGA CDS and the CTR. A linear regression model (dashed line) is fitted across all six panels. The regression equation with R2 value and the 95% confidence interval is embedded in the plot (grey shade).
