# Supplementary material for: Cross-oncopanel study reveals high sensitivity and accuracy with overall analytical performance depending on genomic regions
Source: Genome Biol. 2021 Apr 16;22:109. doi: 10.1186/s13059-021-02315-0 (PMC8051090; doi:10.1186/s13059-021-02315-0)
Supplement: Supplementary file 4 — Additional file 4. Review history. [file 13059_2021_2315_MOESM4_ESM.docx]

Review History

**First round of review**

**Reviewer 1**

**Are you able to assess all statistics in the manuscript, including the appropriateness of statistical tests used?** Yes, and I have assessed the statistics in my report.

**Comments to author:**

In this article, SEQC2 Oncopanel Sequencing Working Group summarized benchmarking of oncopanel sequencing. They conducted and assessed sensitivity, FP rates and reproducibility considering variant types, VAF ranges and target genomic regions. I think that there are some questions and points that need to be addressed to improve the manuscript as below;

Points;

1. I think the results are a little complicated because the authors used eight different panels and three reference materials and analyzed the data from various direction. They should summarize the best practices for oncopanel sequencing in the end of the Results or Discussion section for some cases.

2. P15 L15: How did the authors determine "a prescribed depth of coverage" for each library? Please describe that because the depth generally affect the VAF calculation.

3. The authors used KP and KN for calculating sensitivity and FP rates. For more understanding the results, readers need to have detailed information about KP and KN variants. The authors should add a short summary of the variants used in this study even if the information was reported in a companion paper.

4. P17 L17: Please describe the reason why "TFS chose not to test Sample C".

5. P17 L21: I did not understand what means "the increasing known VAF". Please clarify the meaning.

6. The authors indicated that the cross-lab reproducibility is lower and the FP rate is higher outside of the CTR. They must add the discussion about the differences between within and outside of CTR affecting the reproducibility and the FP rates.

7. TMB and VAF distributions may be different among cancer subtypes. I recommend that the authors add a discussion about cancer subtypes in the evaluation of TMB estimation using TCGA data (Fig. 5c and 5d).

8. I think that many researchers extract various information such as mutational signature, intra-tumor genetic heterogeneity and so on when they perform sequencing analysis of cancer genomes. Can they conduct such analyses by only considering high confidence coding regions using panel sequencing? I recommend that the authors would mention additional application of oncopanels in addition to TMB estimation.

**Reviewer 2**

**Are you able to assess all statistics in the manuscript, including the appropriateness of statistical tests used?** Yes, and I have assessed the statistics in my report.

**Comments to author:**

The authors describe the first use of the new benchmarks described in the companion paper, which is really useful to see for a new reference sample like this. The paper would benefit from less subjective and more quantitative and statistically meaningful claims, as noted below. It also will be important to have some curation/confirmation to ensure both the false positives and false negatives being reliably identified as errors in each callset. Otherwise, I expect this will be a valuable companion paper if the points below are addressed:

1. In the title, "high sensitivity and accuracy" is too subjective, since the required sensitivity and accuracy will be different for different applications.

2. In the abstract, the authors say "consistently high sensitivity (> 95%)", which is less useful than giving the range of observed sensitivities. The phrase "consistently high" is subjective and should be removed.

3. The authors state: "Overall, sensitivity across all VAF ranges in all panels was high, generally above 90%, even for the lowest VAF range tested (1%-2.5%). Sensitivity was generally above 95% for VAF above 2.5% and most panels approached 100% sensitivity for VAF greater than 5%." Words like "high", "generally above", and "approached 100%" are not statistically meaningful. It would be better to give confidence intervals around numbers.

4. The authors state: "We initially anticipated similar sensitivity for the Spike-in sample in the VAF range of 2.5-10%, as the variants were designed to be 5% VAF. However, this was not observed, likely due to interference of the Spike-in sample with primers, DNA capture, or variant calling. Custom filtering of the Spike-in's variants was required to derive sensitivity estimates (see Methods)". This contradicts many previous interlab studies of spike-in DNA, so the authors should clearly give the support for this claim if they have it. Alternatively, could it be related to the complex aneuploid background, or due to the spike-in variants being more difficult to detect?

5. Again, the use of "generally low" is not statistically meaningful in: "All panels had generally low FP rates in the CTR, and the FP rates dropped as the VAF cutoff increased (Fig. 2b).

6. The authors state: "We also assessed both intra- and cross-lab reproducibility for variant calls in the CTR with VAF between 2.5% and 20%, and observed that all panels performed well according to this metric (Fig. 2c, Fig. S1a), with cross-lab reproducibility being slightly worse than intra-lab reproducibility in a few instances." Again, the use of "performed well" is not statistically meaningful.

7. The authors state: "Results for Sample A and Sample C largely agreed. Differences may be driven by VAF reporting boundary effects, as Sample C has more variants in the lower VAF ranges (thus near the VAF reporting thresholds for several panels) (Fig. S1b)." Again, the use of "largely agreed" is not statistically meaningful.

8. For the violin plots in Figs 2c, 3a, 4a, 4c, it is important to know whether the number of points that went into the plot are sufficient to show reliable distributions. Their appearance suggests the distributions are unreliable, and that it would be better to show these as dot plots (e.g. http://www.sthda.com/english/wiki/ggplot2-dot-plot-quick-start-guide-r-software-and-data-visualization )

9. The authors state: "Method 2 marked any call in the normal Sample B with VAF between 1% and 10% as a false positive ("B_low", Fig. S3b) due to strong evidence of this sample having normal diploid cell characteristics[25]". All cell lines have some mosaic variants due to cell line drift, even if they are diploid. Could you at least state this as a limitation?

10. The authors state: "Taken together, these results suggest that variant location may drive high cross-lab reproducibility seen in Fig. 2c. Since sensitivity didn't drop noticeably outside the CTR (Fig. 3c), the observed drops in reproducibility (Fig. 4c) may be driven by more FPs outside of the CTR (Fig. 4a)." Could the authors look, for example, if many of the irreproducible variants are in difficult to map regions or homopolymers/tandem repeats?

11. The authors state: "We observed that these panels detected known small indels or MNVs with similar sensitivity and reproducibility as SNVs (Fig. 3a, Fig. S2), but we have less confidence in making the same inference for non-SNVs due to the limited number of known non-SNVs in the reference material." It seems unlikely to be true that MNVs and indels are detected as well as SNVs given many previous papers. If the authors can't statistically support this claim, then they shouldn't make it.

12. The authors state: "Our raw data will be made public to enable further evaluation of various bioinformatics pipelines by the community." Are the samples consented such that the data can be made public?

13. The authors state: "In summary, all eight panels demonstrated good sensitivity for even low VAF variants" Again, the use of "good sensitivity" is not statistically meaningful.

14. The authors state: "Briefly, CTR is the overlap of targeted regions of four whole exome sequencing panels, UCSC coding genes, Ensemble exons, and NIST high confidence genomic regions, followed by a removal of low complexity regions[25]." it would be helpful to readers to describe the limitations of the CTR, such as ignoring difficult regions and places with systematic sequencing errors, so that FP rates are likely much higher

15. The authors state: "The simulation aimed to reduce the variant density and reduce low VAF variants to better mimic a "real world" clinical sample." Could the authors say more about why this was necessary here and not for the variant studies? I suspect it is because the FP rate has a more realistic effect if the variant density is realistic.

16. Did the authors manually curate a subset of the putative false positives and false negatives to ensure the benchmark is reliably identifying errors in each callset and that the performance metrics can be believed? This validation is an integral part of demonstrating widely used benchmarks like https://rdcu.be/b4UMa and https://doi.org/10.1101/2020.07.24.212712

**Authors Response**

**Point-by-point responses to the reviewers’ comments:**

Reviewer #1

In this article, SEQC2 Oncopanel Sequencing Working Group summarized benchmarking of oncopanel sequencing. They conducted and assessed sensitivity, FP rates and reproducibility considering variant types, VAF ranges and target genomic regions. I think that there are some questions and points that need to be addressed to improve the manuscript as below;

1. I think the results are a little complicated because the authors used eight different panels and three reference materials and analyzed the data from various direction. They should summarize the best practices for oncopanel sequencing in the end of the Results or Discussion section for some cases.

*We appreciate the reviewer’s excellent suggestion. As a summary of major findings, a text table (Box 1) of best practice guidelines for oncopanel sequencing has been added at the end of introduction.*

2. P15 L15: How did the authors determine "a prescribed depth of coverage" for each library? Please describe that because the depth generally affect the VAF calculation.

*Each panel provider determined the depth of coverage. The related sentences have been revised Revised in Results: “Libraries were sequenced on the same sequencing platform for each panel and the panel provider then performed panel-specific variant calling.” Addition in Online Methods: “Each panel provider determined the depth of coverage per library and Sample C libraries were sequenced twice as deep as other samples.”*

3. The authors used KP and KN for calculating sensitivity and FP rates. For more understanding the results, readers need to have detailed information about KP and KN variants. The authors should add a short summary of the variants used in this study even if the information was reported in a companion paper.

*We appreciate the reviewer’s suggestion. In addition to the brief summary of the variants in the beginning of subsection “Overview of study design” and variant distribution by VAF in Figure 1, we provided more details at the end of the subsection.*

*“More than 42,000 KPs and 10 million KNs were characterized in Sample A in the 22 Mb high confidence exon coding regions through extensive sequencing of Sample A and the individual cell line DNA samples that made up Sample A. KN loci had the mismatch error rate below 0.25%. The distribution of KPs by VAF is shown in Fig. 1a.”*

4. P17 L17: Please describe the reason why "TFS chose not to test Sample C".

*TFS has a clear VAF threshold for variant calling at 2.5%. Sample C was equal mass mixture of Sample A and B. Assuming the variant is not present in Sample B, its VAF in Sample C was reduced by half. Since the majority of low VAF (between 1% and 20%) variants in Sample C have VAF below 2.5%, TFS made the request to skip Sample C. Another reason could be cost cutting as all reagents and sequencing at the test sites were funded by the panel providers. Sample C required twice the depth of sequencing. It might complicate the sequencing plan by including Sample C. The sentence has been revised as “Sample C was not tested by TFS.” It has been moved up to the beginning of the subsection where the testing samples were mentioned.*

5. P17 L21: I did not understand what means "the increasing known VAF". Please clarify the meaning.

*Thanks for pointing out this. It has been revised to “known variants of higher expected VAFs.*

6. The authors indicated that the cross-lab reproducibility is lower and the FP rate is higher outside of the CTR. They must add the discussion about the differences between within and outside of CTR affecting the reproducibility and the FP rates.

*We appreciate the reviewer’s suggestion. One paragraph in the Discussion section has been expanded accordingly.*

*“To understand the impact of genomic region on sensitivity, reproducibility, and FP rates, we assessed all variants compared to those only within the CTR. Sensitivity was consistent for each panel between the CTR and HC_CR (Fig. 3c). However, because known variants were only available within HC_CR, we were not able to assess sensitivity for the regions outside of HC_CR. We hypothesized that sensitivity would be the same outside of HC_CR or slightly lowered. As for the FP rate, we observed increases of multiple folds for all panels outside of the CTR when studying the impact of genomic region (Fig. 4a). Reproducibility was lower outside of the CTR (Fig. 4c). A plausible inference would be that the much higher FP rate outside of the CTR led to lower reproducibility in that region. “Real world” clinical samples likely harbor much fewer low VAF variants (VAF < 20%) than Sample A or C that was used here. With fewer variants, the effect of FP rate on reproducibility will be even more pronounced, i.e., the change in reproducibility will be greater between the CTR and outside of the CTR. In the extreme case of samples without any low VAF variants, e.g., a pure sample from a healthy infant, reproducibility will be completely dependent on the FP calls with no relationship to sensitivity. When reproducibility would play an important role in the specific application of oncopanel sequencing, for example, profiling multiple samples to study intra-tumor genetic heterogeneity, one may consider restricting the analysis to the CTR.”*

7. TMB and VAF distributions may be different among cancer subtypes. I recommend that the authors add a discussion about cancer subtypes in the evaluation of TMB estimation using TCGA data (Fig. 5c and 5d).

*We agree that TMB and VAF distributions vary across cancer subtypes. Within this study we considered VAF and TMB thresholds from the analytical perspective, without reference to specific cancer subtypes. Whilst we anticipate that the technical relationship between TMB and technical variance (as shown in Figs. 5c and 5d) would remain independent of cancer subtypes, we would expect that the cancer subtype may inform the selection of oncopanel and VAF threshold to optimize diagnostic yields. Therefore, we have included the following discussion about cancer subtypes as follows:*

*“Within this study we have evaluated the impact of analytical variables on the detection of mutations and TMB estimation without reference to specific cancer subtypes. Whilst the described relationships between analytical variables and TMB estimation are likely to remain constant regardless of cancer subtypes, the cancer type may inform the selection of oncopanel and VAF threshold to optimize diagnostic yields. For example, greater mutation density, which is typical in lung cancers and melanomas, will enable a more confident estimation of TMB with a lower overall CVs (as shown in Fig. 5d for high TMB values). Therefore, a moderately sized oncopanel with a higher VAF threshold may be sufficient. Alternatively, lower VAFs, which confound confident diagnosis, are typical in cancers with lower sample purity or greater intratumor heterogeneity, such as pancreatic cancers. In these cancers, a larger oncopanel with a lower VAF threshold may be required to confidently estimate TMB.”*

8. I think that many researchers extract various information such as mutational signature, intra-tumor genetic heterogeneity and so on when they perform sequencing analysis of cancer genomes. Can they conduct such analyses by only considering high confidence coding regions using panel sequencing? I recommend that the authors would mention additional application of oncopanels in addition to TMB estimation.

*We appreciate the reviewer’s constructive suggestion. We add intra-tumor genetic heterogeneity to the discussion about reproducibility and FP rate. Please see our response to Point 6 above. Since most mutational signature analyses have been done with WGS and WES data, it would be interesting to examine the question whether large oncopanels would be able to provide an alternative approach. We fear that any discussion without a lengthy evaluation may be insufficient or even misleading to the readers.*

Reviewer #2

The authors describe the first use of the new benchmarks described in the companion paper, which is really useful to see for a new reference sample like this. The paper would benefit from less subjective and more quantitative and statistically meaningful claims, as noted below. It also will be important to have some curation/confirmation to ensure both the false positives and false negatives being reliably identified as errors in each callset. Otherwise, I expect this will be a valuable companion paper if the points below are addressed:

1. In the title, "high sensitivity and accuracy" is too subjective, since the required sensitivity and accuracy will be different for different applications.

*We understand the reviewer’s perspective and can propose another title, “Crossoncopanel study evaluates sensitivity and accuracy with overall analytical performance depending on genomic region”. However, we would contend that the descriptive title is less informative as all evaluated oncopanels have clearly demonstrated high sensitivity (>99% for variants with VAF greater than 5%) and accuracy (i.e., low FP rates of approximately 1 FP per Mb or less for VAF greater than 5%) in the high confidence coding region. Such key findings are of great interest to the readers. We leave to the editor’s discretion whether it is appropriate to retain the original title.*

2. In the abstract, the authors say "consistently high sensitivity (> 95%)", which is less useful than giving the range of observed sensitivities. The phrase "consistently high" is subjective and should be removed.

*We appreciate the reviewer’s drive for rigorousness. We have revised the sentence and the subsequent one to be more precise.*

*“The study revealed that all panels demonstrated high sensitivity (96.5% - 100%) across targeted high confidence coding regions and variant types (single nucleotide variants (SNVs) vs. small indels or multi-nucleotide variants (MNVs)) for the variants previously verified to have variant allele frequency (VAF) in the 5-20% range. Sensitivity was reduced by utilizing VAF thresholds due to inherent variability in VAF measurements. With a VAF threshold of 2.5%, sensitivity ranged from 84.2% to 95.9% across panels for variants of expected VAF within 2.5-5%.”*

3. The authors state: "Overall, sensitivity across all VAF ranges in all panels was high, generally above 90%, even for the lowest VAF range tested (1%-2.5%). Sensitivity was generally above 95% for VAF above 2.5% and most panels approached 100% sensitivity for VAF greater than 5%." Words like "high", "generally above", and "approached 100%" are not statistically meaningful. It would be better to give confidence intervals around numbers.

*We appreciate the reviewer’s suggestion. The sentences have been revised with confidence intervals provided in a supplementary table.*

*“Overall, sensitivity across all VAF ranges in all panels was high with narrow confidence intervals (Supplementary Table 2). It ranged from 87.1% to 98.3% for the lowest VAF range (1-2.5%) tested by AGL, BRP, IGT, and QGN (Fig. 2a). Sensitivity was greater than 96.8% for the 2.5-5% range and greater than 98.4% for the 5-20% range. The unusual sensitivity for ILM in Sample C for the 10-20% range was due to a false negative. Other exceptions were ILM, ROC, and TFS for the 2.5-5% range, as their variant calling pipelines had a VAF reporting threshold very close to 2.5%. So their sensitivity was reduced due to a boundary VAF threshold effect (see below for the effect of VAF cutoff on sensitivity).”*

4. The authors state: "We initially anticipated similar sensitivity for the Spike-in sample in the VAF range of 2.5-10%, as the variants were designed to be 5% VAF. However, this was not observed, likely due to interference of the Spike-in sample with primers, DNA capture, or variant calling. Custom filtering of the Spike-in's variants was required to derive sensitivity estimates (see Methods)". This contradicts many previous interlab studies of spike-in DNA, so the authors should clearly give the support for this claim if they have it. Alternatively, could it be related to the complex aneuploid background, or due to the spike-in variants being more difficult to detect?

*Acrometrix hotspot DNA controls have much more (about 10 times more) variants than other spike-in DNA samples and thus presents a comprehensive picture for spike-in synthetic DNA controls. To address the reviewer’s concern, the passage has been revised slightly to describe the specific filtering of the Spike-in’s variants for IDT, QGN, and TFS. The high variant density round some variants can help explain the low sensitivity observed for ROC. Below is the revised passage:*

*“We initially anticipated similar sensitivity for the Spike-in sample in the VAF range of 2.5-10%, as the variants were designed to be 5% VAF. However, this was not observed, likely due to interference of the nearby Spike-in variants with amplicon primers, DNA capture, or variant calling. To derive sensitivity estimates, custom filtering of the Spike-in’s variants was carried out by the panel providers to exclude variants under primer interference for QGN and TFS or variants in dense clusters for IDT (see Supplementary Methods for details). Likewise, several dense variant clusters led to the unexpected low sensitivity reported for ROC (88.0%).”*

5. Again, the use of "generally low" is not statistically meaningful in: "All panels had generally low FP rates in the CTR, and the FP rates dropped as the VAF cutoff increased (Fig. 2b).

*We followed the reviewer’s suggestion and revised the sentence accordingly.*

*“All panels had FP rates lower than 10.5 per Mb in the CTR with each panel’s default VAF threshold, and the FP rates dropped as the VAF cutoff increased (Fig. 2b).”*

6. The authors state: "We also assessed both intra- and cross-lab reproducibility for variant calls in the CTR with VAF between 2.5% and 20%, and observed that all panels performed well according to this metric (Fig. 2c, Fig. S1a), with cross-lab reproducibility being slightly worse than intra-lab reproducibility in a few instances." Again, the use of "performed well" is not statistically meaningful.

*We followed the reviewer’s suggestion and revised the sentence accordingly.*

*“We also assessed both intra- and cross-lab reproducibility for variant calls in the CTR with VAF between 2.5% and 20% and observed that they were similar for each panel sample combination (Fig. 2c, Fig. S1a), with cross-lab reproducibility being slightly worse than intra-lab reproducibility in a few instances.”*

7. The authors state: "Results for Sample A and Sample C largely agreed. Differences may be driven by VAF reporting boundary effects, as Sample C has more variants in the lower VAF ranges (thus near the VAF reporting thresholds for several panels) (Fig. S1b)." Again, the use of "largely agreed" is not statistically meaningful.

*We followed the reviewer’s suggestion and revised the sentence accordingly.*

*“Results for Sample A and Sample C were similar for VAF above 5% but showed some noticeable differences in the VAF range 2.5-5%. The differences may be driven by VAF reporting boundary effects, as Sample C has more variants in the lower VAF ranges (thus near the VAF reporting thresholds for several panels) (Fig. S1b).”*

8. For the violin plots in Figs 2c, 3a, 4a, 4c, it is important to know whether the number of points that went into the plot are sufficient to show reliable distributions. Their appearance suggests the distributions are unreliable, and that it would be better to show these as dot plots (e.g. http://www.sthda.com/english/wiki/ggplot2-dot-plot-quick-start-guide-r-software-and-data-visualization )

*The typical number of data points for each violin plot is 32 for intra-lab reproducibility (Fig. 2c), 96 for cross-lab reproducibility (Fig. 2c and 4c), 24 for sensitivity estimation (Fig. 3a), or 12 for FP rate (Fig. 4a). Given these large numbers of data points, we would contend that dot plots will be less informative and visually difficult for the same graph area. We made reproducibility plots as dot plots (see attached Possible Suppl Figures Plot #1 and #2 for the corresponding dot plots for Fig. 2c and 4c, respectively) and later replaced them with violin plots. To keep the graph styles consistent, we are hesitant to make dot plots only for FP rate. Another option is to include the corresponding dot plots as supplementary figures where there is no limit on the graph area. We leave this to the editor’s discretion.*

9. The authors state: "Method 2 marked any call in the normal Sample B with VAF between 1% and 10% as a false positive ("B_low", Fig. S3b) due to strong evidence of this sample having normal diploid cell characteristics[25]". All cell lines have some mosaic variants due to cell line drift, even if they are diploid. Could you at least state this as a limitation?

*We appreciate the reviewer’s suggestion. This limitation has been mentioned along with a limitation for Method 3.*

*“Method 2 might overestimate the FP rate by failing to exclude rare mosaic variants caused by cell line drift. Method 3 could underestimate the FP rate by failing to recognize those FP calls due to systematic errors as they would be called in all three samples.”*

10. The authors state: "Taken together, these results suggest that variant location may drive high cross-lab reproducibility seen in Fig. 2c. Since sensitivity didn't drop noticeably outside the CTR (Fig. 3c), the observed drops in reproducibility (Fig. 4c) may be driven by more FPs outside of the CTR (Fig. 4a)." Could the authors look, for example, if many of the irreproducible variants are in difficult to map regions or homopolymers/tandem repeats?

*We agree with the reviewer that it would be helpful to identify the possible contributors for more irreproducible FPs outside of the CTR. We prefer to not include such an analysis for the following reasons. There is no universally accepted mappability score and thus it is difficult to conduct a quantitative analysis. Different panels reported drastically different FP rates outside of the CTR. We feel that dissecting the failure mode of the panels is out of the scope of this study.*

11. The authors state: "We observed that these panels detected known small indels or MNVs with similar sensitivity and reproducibility as SNVs (Fig. 3a, Fig. S2), but we have less confidence in making the same inference for non-SNVs due to the limited number of known non-SNVs in the reference material." It seems unlikely to be true that MNVs and indels are detected as well as SNVs given many previous papers. If the authors can't statistically support this claim, then they shouldn't make it.

*We followed the reviewer’s suggestion and revised the passage accordingly.*

*“We observed that these panels detected the known small indel or MNV positives with similar sensitivity and reproducibility as known SNVs (Fig. 3a, Fig. S2). However, with the limited amount of small indel/MNV positives identified as a proportion of all identified variants in the reference sample (1.2%), it is likely given prior research in the field that these small indel/MNV positives identified in the reference sample are in group that is easier to detect, in general. Therefore, we cannot suggest that these panels find small indels/MNVs with the same proficiency as SNVs throughout their target regions until we have identified a larger and more representative set of indels/MNVs from which to compare.”*

12. The authors state: "Our raw data will be made public to enable further evaluation of various bioinformatics pipelines by the community." Are the samples consented such that the data can be made public?

*We are happy to confirm that the samples consent was obtained, and the data has been deposited to NCBI SRA. All the data will be made public when this manuscript is published. Below is a private link for the editors and reviewers before the public release. https://dataview.ncbi.nlm.nih.gov/object/PRJNA677997?reviewer=duruju76d0n8c5d68 mmc73qhfe*

13. The authors state: "In summary, all eight panels demonstrated good sensitivity for even low VAF variants" Again, the use of "good sensitivity" is not statistically meaningful.

*We appreciate the reviewer’s constructive comment. The sentence has been revised with values for sensitivity.*

*“In summary, all eight panels achieved high sensitivity (ranging from 84.2% to 100%) for variants of low VAF between 1% and 5% (Fig. 2a).”*

14. The authors state: "Briefly, CTR is the overlap of targeted regions of four whole exome sequencing panels, UCSC coding genes, Ensemble exons, and NIST high confidence genomic regions, followed by a removal of low complexity regions[25]." it would be helpful to readers to describe the limitations of the CTR, such as ignoring difficult regions and places with systematic sequencing errors, so that FP rates are likely much higher.

*We appreciate the reviewer’s helpful comment. We have added a brief discussion about the limitations of the CTR and the potential benefits that would be gained through expanding the CTR. The added description is listed below.*

*“Although most pathogenic mutations are within the CTR[25], restricting the analysis of clinical samples to the CTR may lead to the exclusion of certain mutations of interest. Currently, the CTR has ignored difficult regions and places with systematic sequencing errors. Extending the CTR will be a good direction for future work.”*

15. The authors state: "The simulation aimed to reduce the variant density and reduce low VAF variants to better mimic a "real world" clinical sample." Could the authors say more about why this was necessary here and not for the variant studies? I suspect it is because the FP rate has a more realistic effect if the variant density is realistic.

*The reviewer is correct. We have added the following passage:*

*“While the high variant density and high percentage of variants with VAF below 5% in the test samples provided an advantage in estimating detection sensitivity with narrow confidence intervals, they presented obstacles here for the evaluation of TMB technical variability. The high variant density (equivalent to a TMB value about 1000) in the test samples will overshadow the effect of the FP calls and thus greatly underestimate TMB technical variability. The high percentage (over 75% in Sample C) of variants with VAF below 5% may lead to an overestimate of TMB technical variability due to the variability in detecting these low VAF variants.”*

16. Did the authors manually curate a subset of the putative false positives and false negatives to ensure the benchmark is reliably identifying errors in each callset and that the performance metrics can be believed? This validation is an integral part of demonstrating widely used benchmarks like https://rdcu.be/b4UMa and <https://doi.org/10.1101/2020.07.24.212712>

*We appreciate the reviewer’s constructive comments. We first examined the reproducibility of the putative false positives and false negatives in the CTR for each panel. Except very few of them (5 out of about 200 for AGL, 3 out of 11 for IGT) identified by Method 2 (B_low), all putative false positives were not detected in more than 50% of library replicates. Most of them were called in only one library (see attached Possible Suppl Figures Plot #3). All three false positives called by IGT in Sample B were 3 bp deletions. Each was also called in Sample A with a similar number of library replicates and a strikingly similar VAF (the absolute difference was less than 0.1%). They were likely caused by some systematic errors or biases. Similarly, three false positives called by AGL in Sample B were called in Sample A, each with a similar number of library replicates. The absolute differences in VAF were 0.2%, 0.8%, and 0.6%. We feel confident that these false SNV calls were caused by some systematic errors or biases. The other two putative false positive calls may require further investigation. They were called in Sample B with a low average VAF, 1.3% and 2.0%, respectively. They were not called in any Sample A or Spike-in libraries. The Spike-in sample used Sample B as the genomic background. It was sequenced to the same targeted depth as Sample B. If we count all Sample B and Spike-in libraries, these two punitive false positives were called in less than 50% of library replicates. This supports our classification of them as false positives. By the way, the contribution to the FP rate by these two false positives was less than 10%.*

*All missed detections (false negatives) in Sample A or C were due to a combination of low VAF and the panel’s VAF threshold. The only surprise is that ILM missed a known variant in Sample C with expected VAF between 10% and 20%. The known variant was called by large panels such as AGL and BRP. Based on our analysis, our benchmark sets of known positives and negatives in Sample A can be used to reliably identify errors in each panel. This could be added as another topic in discussion. However, the discussion is already quite long. We leave this to the editor’s discretion.*

**Second round of review**

**Reviewer 1**

The authors have addressed my concerns.

**Reviewer 2**

The authors have done a good job of responding to the reviewers' comments, making more quantitative claims and adding a good discussion of the limitations of the work, particularly in excluding more challenging regions. I have only a couple follow-up points to be addressed:

1. I recommend the authors include the discussion about curation of FP and FN at least as supplementary information, and add the main points to the results.

2. The SRA link did not work for me, so make sure it is made fully public before publication.

3. The authors state "Ethics approval and consent to participate" and "Consent for publication" are "Not applicable". According to Genome Biology policy, I understand the manuscript needs to state what Ethics body made this determination, particularly because these are human cell lines for which the authors are making available public genome sequencing data, which should have the appropriate consent.
